# NEONATAL DEATH IN INDIA: BIRTH ORDER IN A CONTEXT OF MATERNAL UNDERNUTRITION*

**DOI:** 10.1093/ej/ueab028

**Published:** 2021-04-15

**Authors:** Diane Coffey, Dean Spears

**Affiliations:** Department of Economics, University of Texas at Austin, Austin, Texas 78712, USA.

## Abstract

We document a novel fact about neonatal death, or death in the first month of life. Globally, neonatal mortality is disproportionately concentrated in India. We identify a large effect of birth order on neonatal mortality that is unique to India: later-born siblings have a steep survival advantage relative to the birth-order gradient in other developing countries. We show that India’s high prevalence of maternal undernutrition and its correlation with age and childbearing can explain this pattern. We find that Indian mothers exit the underweight body mass range at an internationally comparatively high rate as they progress through childbearing careers.

The rapid human development of the past decades is reflected in a steep fall in infant mortality. In 1960, 12% of babies worldwide died in their first year of life compared to only 3% in 2015.^1^ Over three-fifths of the infant deaths that still occur are neonatal deaths, meaning deaths in the first month of life. Although progress in reducing both neonatal and infant deaths has been rapid, it has also been uneven. Twenty-seven percent of neonatal deaths now occur in India. India has a large population, but its share of neonatal mortality is disproportionately large: it is home to 19% of worldwide births.

Why is neonatal mortality in India so disproportionately large? Investigating neonatal mortality (NNM)^2^ in India, we document a novel fact. We identify a unique later-born NNM advantage in India. We exploit data on over six million births in the developing world to describe the relationship between birth order and mortality, following the econometric methods of Blake (1989) and Black et al. (2005). This empirical strategy allows us to separate birth order from other correlates of fertility in India that differ from other developing countries (Vogl, 2016). The gradient that we estimate is quantitatively large and is specific to NNM in India: there is no similarly large later-born advantage for NNM in the rest of the developing world, nor is there an internationally unusual later-born advantage for post-neonatal mortality (death between months 2–11) in India. This poses a puzzle: why is NNM in India higher for early-born siblings than for later-born siblings? This result, as we show, is not explained by differences in medical care at birth.

We provide evidence that India’s later-born NNM advantage reflects a pernicious intersection of maternal nutrition and women’s social status in India (Das Gupta, 1995). Neonatal death is substantially influenced by birth weight and intrauterine growth. Mothers in India are especially likely to be underweight: as Coffey (2015b) showed, over 40% of women in India are underweight at the beginning of pregnancy (in the sense of low body mass relative to height). Moreover, as we document, the prevalence of being underweight in India—in contrast with other developing populations—is particularly concentrated among young women at the beginning of their childbearing careers. As women in India age, have children and gain social status, they become less likely to be underweight.

To explain India’s unique later-born NNM advantage, we document these facts about maternal underweight for India, and show that they differ compared with the rest of the developing world. We use two strategies to show that the fraction of a cohort of mothers who are underweight declines as they age more steeply in India than elsewhere: first, by matching cohorts across repeated cross-sectional survey waves, and second, in a specialised longitudinal survey that tracked a sample of mothers in four developing countries.

The effects that we document are large. In fact, the pattern we document is large enough to be an important component of worldwide average NNM. This is in part because the effects that we find are large, and in part because India contributes a large fraction of global NNM. In the final part of the paper, we compute a projection of how different India’s overall neonatal mortality rate would be, in the absence of its unique birth-order pattern (but with other correlations held equal). To do this, we ask how many Indian neonates would die if India’s earlier-born disadvantage were only as large as the earlier-born disadvantage in other developing countries. Changing India in this hypothetical way would eliminate, in an accounting sense, about 8% of all neonatal deaths worldwide. So, the links among maternal undernutrition, women’s social status and neonatal death in India should be a priority for human development policy.

## Contributions to the Literature

The facts presented in this paper make several contributions throughout empirical economics. First, correlates of birth order have received continued attention in labour economics and economic demography (Behrman and Taubman, 1986). Because of binding requirements for complete data that can separate sibsize from birth order, this literature has generally focused on developed countries where population-level administrative and vital records are available. No prior study has examined birth order in the developing world with such a large dataset, with so many mothers who have completed fertility.

In the developed-country literature on birth order and child outcomes, a common result is that later-born siblings are at a disadvantage. For example, Black et al. (2005) used a rich dataset that contains information on the entire population of Norway to show that later-born siblings attain less education.^3^ In contrast, we find that later-born siblings are at an especially large neonatal mortality *advantage* in India because (unlike in other contexts) mothers’ nutrition is a sharply restricted input to child health that improves over a childbearing career, reflecting improvements in social status. In particular, although we show that later-birth-order children are somewhat more likely to survive infancy throughout the developing world,^4^ they are quantitatively even more likely to survive infancy in India. Furthermore, we document an unusually large average disadvantage of children born to higher-fertility mothers in India relative to children born to higher-fertility mothers in other countries. In India, a large *disadvantage* to high *sibsize* co-exists with a large *advantage* to later *birth order*. This finding reinforces a methodological literature that emphasises the endogeneity of sibsize for estimating effects of birth order (Blake, 1989; Spears et al., 2019).

Second, the large and economically important puzzles of early-life health in India have received sustained attention at the frontiers of development economics (Deaton, 2013). This large literature includes both studies of differences between India and other parts of the world (Tarozzi, 2008; Bhagwati and Panagariya, 2012; Drèze and Sen, 2013; Headey et al., 2015; Jayachandran and Pande, 2017; Spears, 2018) as well as puzzling differences within India in health or nutrition between demographic groups or across time (Deaton and Drèze, 2009; Bhalotra et al., 2010; Eli and Li, 2013; Coffey and Spears, 2018).

Third, perhaps most broadly, we advance a literature documenting the continuing relevance of family structures for outcomes in developing countries (Strauss and Thomas, 1995), where state safety nets are less developed and incomplete markets make families an important unit of economic and social organisation. Some of this research documents effects on demographic and health outcomes (Vogl, 2013; Barcellos et al., 2014; Coffey et al., 2017) while other research studies labour market and economic consequences (Foster and Rosenzweig, 2002; Bertrand et al., 2003; Field and Ambrus, 2008; Ardington et al., 2009).

Fourth, maternal nutrition is of policy importance (World Health Organization, 1995; Alderman and Behrman, 2006). Economists increasingly recognise that what happens in very early life matters for later-life health and human capital (Maluccio et al., 2009; Almond et al., 2017). This is especially true in developing countries, where early-life insults are severe and varied, and fewer opportunities exist for remediation (Spears, 2012; Currie and Vogl, 2013).

Finally, the descriptive facts we document about NNM and birth order are of such quantitative magnitude to themselves to be an important contribution to understanding patterns of infant death globally. Thus, our results join other recent studies in economics that have documented important novel facts about mortality (Case and Deaton, 2015; Chetty et al., 2016). For comparison, Chen et al. (2016) have recently studied the large infant mortality rate (IMR) difference between the United States and Europe. Combining their estimates with standard quantification of the value of a statistical life, they compute that ‘reducing the US IMR to that of Scandinavian countries would be worth on the order of US$84 billion annually. By this metric, it would be ‘worth it’ to spend up to $21,000 on each live birth to lower the infant mortality risk to the level in Scandinavia.’ Mortality rates are much higher in developing countries, where many more babies are born. The excess NNM that we newly document between India and the rest of the developing world is about three times as large as the important IMR gap that they study between the United States and Europe, expressed as a rate, and applies to many more births.^5^

## Outline

In Section 1 we present the background: India is a country with very high neonatal mortality, despite the fact that a large and increasing fraction of births take place in hospitals rather than at home. India’s population is also a population where maternal nutrition is very poor on average, but improves as women gain status over a childbearing career. A large literature in labour economics, especially in developed countries, has identified human capital disadvantages of being a later-birth-order child. However, India’s pattern of maternal nutrition gives reason to expect an opposite result in the Indian context. Motivated by these facts, we estimate an effect of birth order on neonatal mortality, using Demographic and Health Survey data introduced in Section 2 and an empirical strategy described in Section 3.

In Section 4 we present our main results, first as descriptive summaries of early-life mortality by birth order and sibsize, and then as a series of regression results that highlight the value of our empirical strategy. The association between birth order on neonatal mortality in India is not reversed by an opposite association with post-neonatal mortality (as might have happened if the effect merely accelerated a set of deaths that were otherwise likely to occur). We also verify that our results are not driven by medical care: birth in a hospital, rather than at home, is a weak predictor of neonatal mortality in India (Coffey, 2019). Although high-quality intensive medical care of low-birth-weight infants has been shown to reduce neonatal mortality (Paneth et al., 1982), the average quality of medical care that births in our Indian sample would have received is very low (Chaudhury et al., 2006; Das et al., 2008; Coffey, 2014).

In Section 5 we present a collage of evidence that maternal nutrition can account for our main result. We estimate longitudinal weight gain of cohorts of women by matching cohorts at different ages across successive Demographic and Health Surveys. We find that more mothers exit the dangerously underweight range of body mass as they age in India than in other places. This is in part because more women in India are underweight to begin with. We then briefly note a separate panel data source that is consistent with these facts holding longitudinally for individual mothers. Finally, in a comparison across Demographic and Health Surveys in various countries, we find that those survey rounds where there is a steeper negative age gradient between weight and underage are those where later birth order has a less positive (or more negative) association with NNM, on average.

In Section 6 we consider the magnitude of our estimates in an international comparison. We use a hypothetical demographic decomposition to summarise and scale our estimates. This computation projects that global NNM would be substantially lower if India shared the rest of the developing world’s birth-order pattern.

## 1. Background on Maternal Nutrition, Birth Order and Early-Life Health in India and the Developing World

Compared with other developing countries, neonatal mortality in India is unusually common. Our estimates from 2015 data find that 27% of neonatal deaths happen in India, which is essentially identical to what Lawn et al. (2005) found for 2000. A large fraction of India’s NNM occurs in the state of Uttar Pradesh, which has a population as large as Brazil’s (over 200 million people) and a neonatal mortality rate far greater than the rest of India.^6^ In general, variation in NNM across places and times can be largely explained by poor healthcare at birth and by low birth weight (Bhutta et al., 2008).

Improving rates of institutional delivery—meaning causing births to happen in medical facilities rather than in homes—is typically presented in policy and epidemiological literatures as the solution for neonatal death in the developing world.^7^ However, over the past decade, the rapid expansion of institutional delivery in India due to a government conditional cash transfer program has not had a large effect on neonatal survival. Between 2005–2006 and 2015–2016, institutional delivery increased from 39% to 79%, but neonatal mortality fell only from 37 in 2003 to 28 in 2013.^8^ In Subsection 4.3 we find that children who are born in hospitals are not less likely to die in neonatal deaths than those born at home. Although these outcomes may surprise policy makers, they are consistent with theories and evidence in the economics literature about poorly monitored public spending programs (Hanushek, 2003; Banerjee et al., 2008; Pritchett, 2009). Health care facilities in this context do not have much incentive to promote health (Coffey, 2014).

Unfortunately, there are no reliable national statistics on birth weight in India. However, national estimates of maternal undernutrition suggest that the prevalence of low birth weight is very high (Coffey, 2015b). Maternal nutrition is important for birth weight and babies’ outcomes (Hytten and Leitch, 1964; Yaktine and Rasmussen, 2009). Low weight gain in pregnancy by mothers causes low birth weight (Ludwig and Currie, 2010), and low-birth-weight babies are less likely to survive early infancy (Almond et al., 2005). Maternal nutrition is especially bad in India due the low social status of young women (Jeffery et al., 1988; Palriwala, 1993). But, as the demography literature implies (Das Gupta, 1995), and as we document in Section 5, women’s social and nutritional statuses improve over a childbearing career, on average.^9^

To learn about the implications of these patterns of maternal status and nutrition, we turn to identifying an effect of birth order, building upon an accomplished literature in labour economics and economic demography. We employ a standard empirical strategy that isolates birth order by controlling flexibly for each count and combination of siblings. As this literature has shown, this strategy requires large, complete data sources that allow researchers to separate birth order from sibsize, cohort and age (Blake, 1989). As a result, much of this literature has focused on developed countries, where in some cases administrative demographic records are available (e.g., Black et al., 2005). A smaller set of papers has investigated birth order in developing countries (Behrman, 1988; Horton, 1988; Emerson and Souza, 2008; De Haan et al., 2014).

A typical conclusion in the birth-order literature is that earlier-birth-order children are better off. As Lehmann et al. (2016) summarised: ‘A growing number of studies find that birth order affects educational attainment and labour market outcomes: younger siblings within the same family have consistently worse adult economic outcomes than their elder siblings.’ Many of these papers are motivated by a theory of parental or household allocation of resources among siblings, such as in a quantity-quality trade-off decision. We find the opposite birth-order effect: later-born siblings in India are more likely to survive the neonatal period than earlier-born siblings. However, our finding is compatible with the prior literature, because a different mechanism is likely to be at work in the context we study: a pattern of maternal undernutrition that is unusual both in its structure and magnitude. Because this determinant of mortality reflects factors operating before birth, we propose that our results are due not to unequal treatment of *children* who are siblings, but rather are due to decreasing severity of neglect of *mothers* at different points in their childbearing careers.

## 2. Demographic and Health Survey Data

As Chen et al. (2016) wrote in the introduction to their study on infant mortality in the United States and Europe, ‘a key constraint on past research has been the lack of comparable micro-datasets across countries. Cross-country comparisons of aggregate infant mortality rates provide very limited insight.’ This constraint is even more binding in the study of developing countries, where credible vital registration systems have often been absent and where census and other official data sources can be limited by both state capacity and incentives (Mehta, 1969; Setel et al., 2007).

We overcome this constraint by using birth histories from 169 Demographic and Health Surveys (DHSs). DHSs are cross-sectional surveys in developing and middle-income countries, organised and funded by USAID. For almost three decades, the DHSs have asked a comparable set of questions to representative samples of adult women of reproductive age. Each DHS round is a cross-sectional survey, but the survey includes a retrospective birth history that collects information on each child ever born alive to the mother. The survey module records the month of birth of the child, whether it died and the age in months at death. These questions are uniform across survey rounds and countries.

Applying DHS data to our question has several advantages. First, the retrospective nature of the birth histories allows us to use empirical strategies for studying longitudinal data, such as mother fixed effects that focus on variation across siblings. Second, because DHS rounds are repeated cross sections, we can separate birth order and sibsize from the passage of historical time and from the cohort of the mother’s and child’s birth. Third, the richness of our large child-level data, observing all births to the mother by the time of the survey, permits us to meet the data requirements necessary to separate birth order from sibsize (Blake, 1989), as well as to link anthropometry and other observables at a much finer level than would be possible with aggregate data.

Our ‘main DHS sample of births’, used to generate the results in Section 4, includes over six million births. Because we are particularly interested in understanding early-life mortality in India, we compare children in India with children elsewhere in the developing world. For the ‘rest of the developing world’, we use an inclusive set of all available DHS rounds in which maternal anthropometry is measured.^10^ We also include births from all three DHSs conducted in India, even though the 1992–1993 Indian DHS lacks maternal anthropometry data and is therefore absent from the analyses in Section 5. Table A.1 in the Online Appendix lists the country and year of all of the 169 DHS rounds used in our paper. Twelve percent of our observations (about 800,000) are from India.

In robustness checks of our main results, we replicate our findings using a restricted sample of almost three million births to mothers whose last birth was at least five years before the survey.^11^ As an additional robustness check, and because the other countries in the DHS likely contain no single proper ‘counterfactual India’ that corresponds exactly with how mortality in India would progress in the absence of the forces we document, we replicate our results comparing India to a sample of DHS rounds from sub-Saharan Africa that has been studied in the economics literature.^12^ DHS rounds in the sub-Saharan African comparison sample are identified in Table A.1 of the Online Appendix.

For most of the analyses in Section 5, we use a ‘women’s anthropometry sample’ that includes all women whose heights and weights were measured by the surveys listed in Online Appendix Table A.1.^13^ Most of the women in this sample are the mothers of children in the ‘main DHS sample of births’ described above, but some are women who have not yet had children. We discuss these data in greater detail in Section 5.

## 3. Empirical Strategy for an Effect of Birth Order

We build upon prior literatures in labour economics and demography that have both (*i*) documented a set of specific, intersecting and often prohibitive challenges and data requirements for identifying an effect of birth order, and (*ii*) established a standard identification strategy for birth order, which we implement here. Blake (1989) described the requirements to identify an effect of birth order: complete data on siblings born in different time periods are needed, such that birth order can be separated from sibsize (or mother’s fertility) and from the child’s and mother’s cohort of birth.

One challenge of identifying effects of birth order is that birth order and sibsize are correlated, in part mechanically: children of high birth order must come from large sibsizes. Another challenge is that later-birth-order children are born to later cohorts, on average. Overcoming these endogeneity concerns requires complete, detailed data on siblings born to mothers of different ages in different cohorts.

Our main data source for infant mortality—the birth histories in the DHSs—offer such longitudinal data, which is collected retrospectively from the mother’s report. The mother’s birth history offers a longitudinal account of *all* of her births by the time of the survey. This allows us to separately account for birth order and sibsize (meaning the total count of births to the mother). This is important to our empirical strategy, because higher sibsize is negatively selective in India to a greater extent than in the rest of the DHSs (Spears et al., 2019). For example, mothers’ height and body mass index (BMI) are *increasing* in sibsize for the average child measured in the rest of the DHSs, meaning that children of higher-fertility mothers are relatively advantaged, on average, in these ways. Mothers’ height and BMI are *decreasing* in sibsize for the average child measured in India.

Our empirical strategy allows sibsize and cohort to have correlations with early-life mortality that are different in India than they are in the rest of the DHSs. This would not be possible for variables that are only measured once per mother at the time of the survey (such as her BMI, or whether her home is in a rural area) or only for a mother’s most recent birth (such as reported indicators of pre-natal and peri-natal health care for the last birth, or anthropometric measures restricted to the youngest children).

We follow Black et al. (2005) in identifying effects of birth order by controlling flexibly for fixed effects by sibsize, and also by showing that results are robust to instead using mother fixed effects. We build upon their method by estimating a *difference*^14^ between the effect of birth order in India and the effect in the rest of the DHSs: we allow each independent variable to be fully interacted with an indicator that the child is from India, rather than the rest of the developing world. Our coefficients of interest, therefore, are the interactions between the India indicator and indicators for birth order:

$${\text{NNM}}_{ims}=\sum_{b}\beta_{1}^{b}{\text{birth order}}_{ims}\times {\mathrm{India}}_{s}+\sum_{b}\beta_{2}^{b}{\text{birth order}}_{ims}\;\;+\sum_{b}\beta_{3}^{b}{\text{sibsize}}_{ms}\times {\text{India}}_{s}+\sum_{b}\beta_{4}^{b}{\text{sibsize}}_{ms}\;\;+f\left({\text{CMC}}_{ims}^{\text{child}},{\text{India}}_{s}\right)+g\left({\text{CMC}}_{ms}^{\text{moth}.},{\text{India}}_{s}\right)\;\;+\gamma_{1}{\mathrm{sex}}_{ims}\times {\mathrm{India}}_{s}+\gamma_{2}{\mathrm{sex}}_{ims}\left[+\alpha_{ms}\right]+\alpha_{s}+\varepsilon_{ims}.$$

Here i, m and s are the numbers of children, mothers and DHS rounds, respectively; b indexes birth order or sibsize; f is the cubic of the child’s CMC^15^ birth cohort; g is the cubic of the mother’s CMC birth cohort; and $\alpha_{ms}$ and $\alpha_{s}$ are the mother fixed and survey round fixed effects, respectively.

Mother fixed effects $\left(\alpha_{ms}\right)$ are in brackets in (1) because we include specifications with and without them. We expect our results to be quantitatively robust and stable when mother fixed effects are added, because their principal anticipated role would be to control for sibsize, which is already controlled for. We will find that our estimates are indeed quantitatively robust to the inclusion or exclusion of mother fixed effects, which suggests that our identification strategy has successfully accounted for potentially biasing heterogeneity across children’s households.

Our main dependent variable is NNM. We also estimate effects on PNM and IMR. IMR, which is the fraction of children who die in the first year of life, is simply the sum of NNM (death in the first month) and PNM (death in months 2–11). In particular, we compare effects on NNM with effects on PNM because PNM typically has different causes than NNM. In India, and in other developing countries, levels of PNM are often determined by the disease environment and the timing and quality of the transition from breastfeeding to other foods (Coffey, 2015a). Because we study *age-specific* mortality rates that are held constant in any dependent variable, our regression equation need not (and cannot) control for child age.

## 4. Results

### 4.1. Summary Mortality Rates

**Fig. 1.**
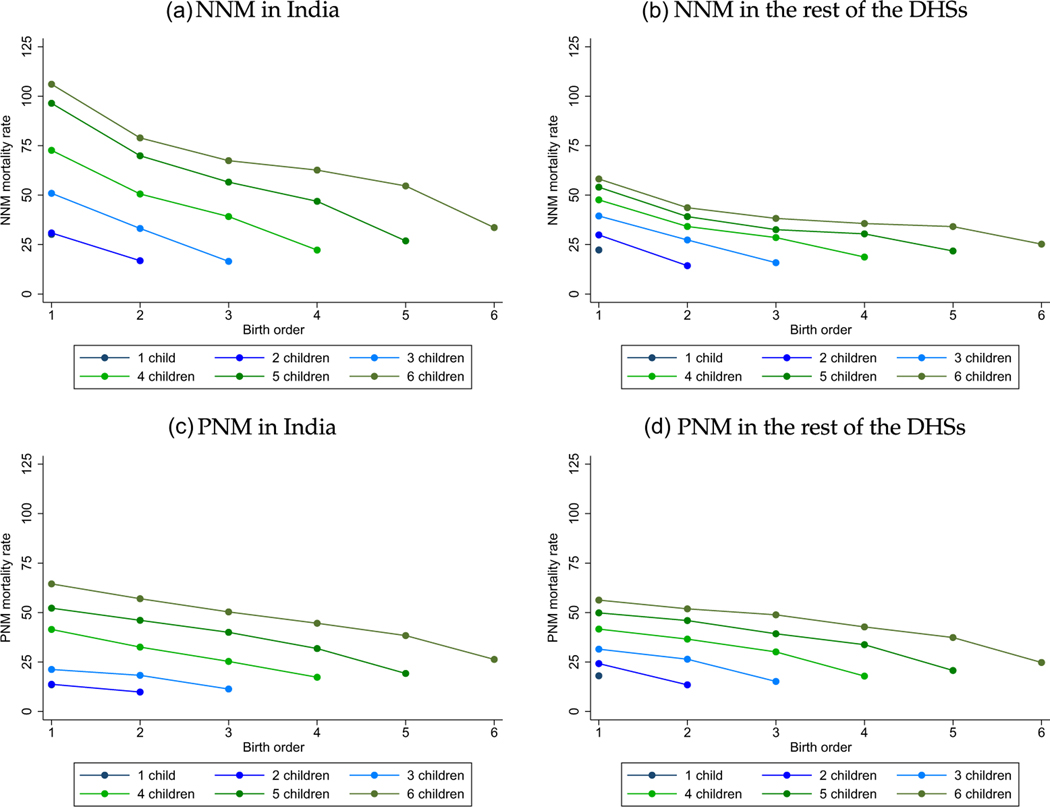
Early-Life Mortality by Birth Order and Sibsize. *Notes:* Data taken from the main DHS sample of births described in Section 2. Mortality rates scaled to per 1,000. For a replication with a restricted sample that excludes mothers with incomplete fertility, see Online Appendix Figure A.1.

In India and in the rest of the developing world, how does the mortality of later-born children compare to that of their earlier-born siblings, on average? We first answer that question with Figure 1, which plots summary mortality rates by birth order and sibsize. These plots in Figure 1, inspired by Blake (1989) and Black et al. (2005), connect mortality rates for successive birth orders with lines within sibsizes.

Two facts are visually apparent. First is the negative selectivity of sibsize, visible in the vertical distance among lines. Sibsize is more negatively selective in India than in the rest of the developing world. That is, children born to higher-fertility mothers are more likely to die than children born to lower-fertility mothers by considerably more in India than in the rest of the developing world. Second is the steep downward slope of the connected lines for NNM in India, indicating a later-born advantage within the same sibsize. Comparing panel (a) for NNM in India with panel (c) for PNM in India, it is clear that India’s later-born IMR advantage (shown in the Online Appendix) is driven by death in the first month of life. Panels (b) and (d) and the Online Appendix show that, although there are downward slopes, in the rest of the DHSs none of these mortality rates are similarly steeply decreasing in birth order. That is, the later-born NNM advantage in India appears unique compared with other developing countries. The neonatal timing of the effect of birth order is strongly suggestive of the mechanisms of maternal nutrition, intrauterine growth restriction and birth weight.

Combining these two conclusions, we see that NNM is highest in India among earlier-born children of mothers who will go on to have many births. Results in the Online Appendix present two robustness checks for this finding. Figure A.1 uses a restricted sample that excludes births to mothers whose most recent birth was within five years of the survey interview. This exclusion is intended to ensure that results are not driven by mothers whose fertility is incomplete at the time of the survey. As a robustness check, and because of a focus in the development economics literature that compares India with sub-Saharan Africa, we further include Online Appendix Figure A.2, which uses the sample described in footnote 12. For both comparisons, the later-born NNM advantage in India is steeper than in other regions by a demographically significant amount.

### 4.2. Main Results

In this section we estimate regression (1). This permits us to test the statistical significance and quantitative robustness of the patterns in Figure 1.

**Fig. 2.**
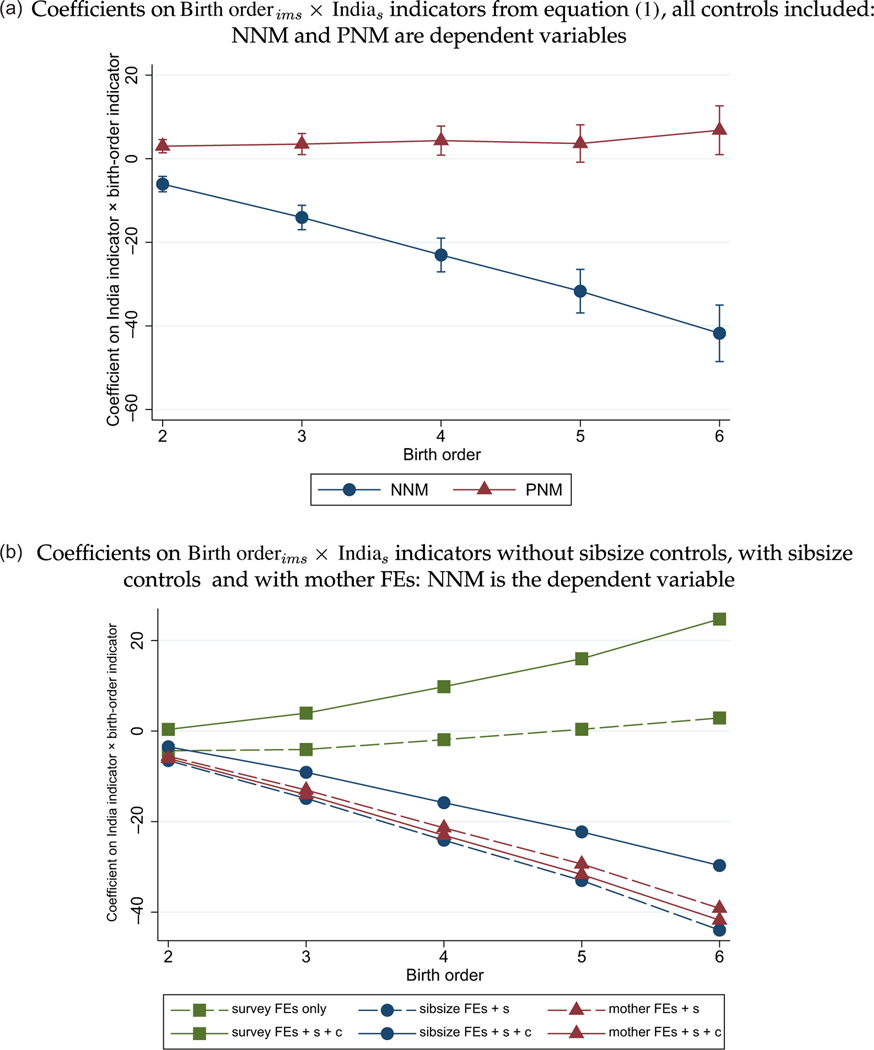
Main Result: How the Relationship between Birth Order and Mortality in India Differs from the Rest of the Developing World. *Notes:* Data taken from the main DHS sample of births described in Section 2. Each connected set of estimates is from a separate regression. The 95% confidence intervals in (a) reflect SEs clustered by survey PSU. Panel (a) uses the fully controlled specification from (1), including mother fixed effects. In (b), s denotes sex and c denotes the birth cohort of the mother and child. For a replication with a restricted sample that excludes mothers with incomplete fertility, see Online Appendix Figure A.3; for a robustness check comparing India with a sub-Saharan African sample, see Online Appendix Figure A.4.

In Figure 2 we plot the regression coefficients on ${\text{birth order}}_{ims}\times {\text{India}}_{s}$ from regression (1), comparing siblings using mother fixed effects. Panel (a) shows our main result: later-birth-order children in India are advantaged in NNM relative to the rest of the developing world. This interaction is specific to NNM; it does not apply to PNM. Moreover, there is no opposite, countervailing effect on PNM, which indicates that the neonatal deaths that we study are not merely an acceleration of infant deaths that would have otherwise happened at a post-neonatal age (that is, not a so-called ‘harvesting’ effect). The effect on survival in the first year is almost the same as the effect on survival in the first month.

Panel (b) investigates the sensitivity of the NNM result to the functional form of (1). It explores the role of sibsize controls by plotting coefficients on birth ${\text{birth order}}_{ims}\times {\text{India}}_{s}$ from a regression that omits them. We expect that omitting sibsize controls will produce qualitatively different results than those of panel (a) because, as we show in Figure 4 (discussed in Section 5), high fertility is a marker of disadvantage in India by more than it is in the rest of the developing world. Panel (b) further explores whether the mother fixed effects specification yields a qualitatively different result than a specification that merely controls for sibsize. We expect that it will not, because, conditional on sibsize, birth order in a complete birth history is unlikely to be correlated with further properties of mothers or households. In these specifications, all controls, including sibsize, are fully interacted with an indicator for the child living in India.

The results of Figure 2(b) verify both of these expectations. Moving from the squares (without sibsize × India) to the circles (with sibsize × India) reverses the sign of the apparent effect of later birth order on NNM. This is consistent with the higher mortality of higher-sibsize children in India (visible in Figure 1), and the mechanical correlation of birth order with sibsize.^16^ Moving from the circles (with sibsize × India) to the triangles (with mother fixed effects, automatically controlling for sibsize) makes no further difference to the estimate of the effect of birth order.

#### 4.2.1. Sex composition of siblings

One important property of mother fixed effects is to control for the *sex composition and pattern* of a child’s siblings.^17^ India is a country where son preference has many demographic consequences (Das Gupta, 1987; Arnold et al., 1998; Kishore and Spears, 2014). One implication is that fertility stopping depends on realised child sex (Clark, 2000). As a result, smaller sibsizes in India contain more boys, on average, than larger sibsizes; moreover, the earlier-born children in a larger sibsize are more likely to be female than male.^18^ Also, sex patterns are correlated with other household properties: families that have a girl and then a boy may be more socially conservative, on average, than families that have a boy and then a girl. Mother fixed effects control for the full realisation of this pattern and make no quantitative difference to our results.

The Online Appendix includes two further investigations of heterogeneity by sex and composition of sibships. The first is to allow sibsize to have different correlations with NNM for boys and girls. Online Appendix Table A.4 shows the results of a regression that adds to (1) sibsize indicators, interacted with an India indicator, interacted with an indicator for the child’s sex. These additional controls mwake no difference to our main result. This result provides an informative contrast with evidence of boys and girls receiving different treatment after birth, when child sex is known (e.g., Barcellos et al., 2014). This therefore points towards a mechanism that operates during pregnancy, before the sex of a child is revealed. The second is to split the sample by whether the first child born to a mother is female or male; see Table A.7. Our main result is present for sibships with both first boys and first girls but is larger for first boys: the later-born neonatal survival relative advantage in India is steeper among sibships with first boys. This is consistent with the interpretation that we introduce in Section 5: a life-course pattern in maternal undernutrition. Kishore and Spears (2014) have shown that women in India who have a first son (rather than daughter) experience relative increases in subsequent social status that is reflected, among other ways, in greater subsequent body mass.

#### 4.2.2. Robustness checks in alternative samples

Table A.2 in the Online Appendix presents regression results with SEs for each of the six specifications shown in Figure 2(b). Table A.3 shows results of the same specifications using IMR, rather than NNM, as the dependent variable. Figure A.3 replicates panel (a) for a restricted sample that omits births to mothers whose last birth was within five years, to ensure that fertility is completed. Figure A.4 replicates this result with the sub-Saharan African comparison sample described in footnote 12. These supplementary analyses confirm the robustness of our main result.

#### 4.2.3. Later-childhood survival

A further question is whether India’s pattern of NNM merely accelerates deaths of infants who would otherwise be likely to die later in childhood. We have already seen evidence against this possibility in the finding that the effect of birth order on IMR is similar to the effect of birth order on NNM. In the Online Appendix, Figure A.5 extends these results using mortality up to two years of age as the dependent variable. Results are very similar to those that use NNM and IMR as dependent variables, indicating that the neonatal deaths documented here are not offset by countervailing effects on later-childhood mortality.

#### 4.2.4. Fertility response to infant death

Any study of the effects of birth order must consider the possibility that fertility endogenously responds to child outcomes, such as neonatal death. If parents replace children who die in early life with additional children who would not otherwise have been born, then late-birth-order children will tend to be born to parents whose children are more likely to die. This is one reason why our mother fixed effects robustness check is important, because it accounts for sibship-level heterogeneity in frailty. In our case, this possibility could only be an important omitted variable in our regressions if the fertility response to neonatal death were large and *different* between India and the rest of the DHSs. In particular, to account for our large interaction-coefficient estimates, the difference between India and the rest of the developing world in this effect would have to be quantitatively very large relative to the variation in fertility rates, because even where neonatal mortality is relatively high, most babies do not die.

In our data, we can observe descriptive statistics that quantify the scope for such a possibility. In particular, we investigate the difference in the probability of having a further birth after births that do and do not survive the neonatal period. The difference between India and sub-Saharan Africa is small and its sign depends on the window studied for subsequent births, suggesting that an important confound is unlikely.^19^

Although these quantities suggest that such an endogenous-fertility threat is implausible in our case, we additionally implement a regression-based robustness check from the birth-order literature. In the Online Appendix, Table A.6 presents results of a robustness test that rules out the possibility that later-born children who are conceived in response to sibling deaths are responsible for our finding. We follow a robustness strategy used by Lundberg and Svaleryd (2017), who studied birth order in Sweden: we exclude any last-born child born after a prior sibling neonatal death. As we show and discuss further in the Online Appendix, our result is robust to this change of the sample.

### 4.3. Medical Care at Birth does not Explain India’s Birth-Order Effect on NNM

Variation in medical care at birth often explains differences across populations in neonatal mortality (Paneth et al., 1982). In India’s most recent DHS, from 2005–2006, 45% of births in the prior five years are recorded to have taken place in a medical facility, rather than at the home of the child’s parents or relatives. Institutional delivery, that is, birth in a medical facility, has been increasing over time in India. Could differences in institutional delivery by birth order account for the birth-order gradient observed in India?

**Fig. 3.**
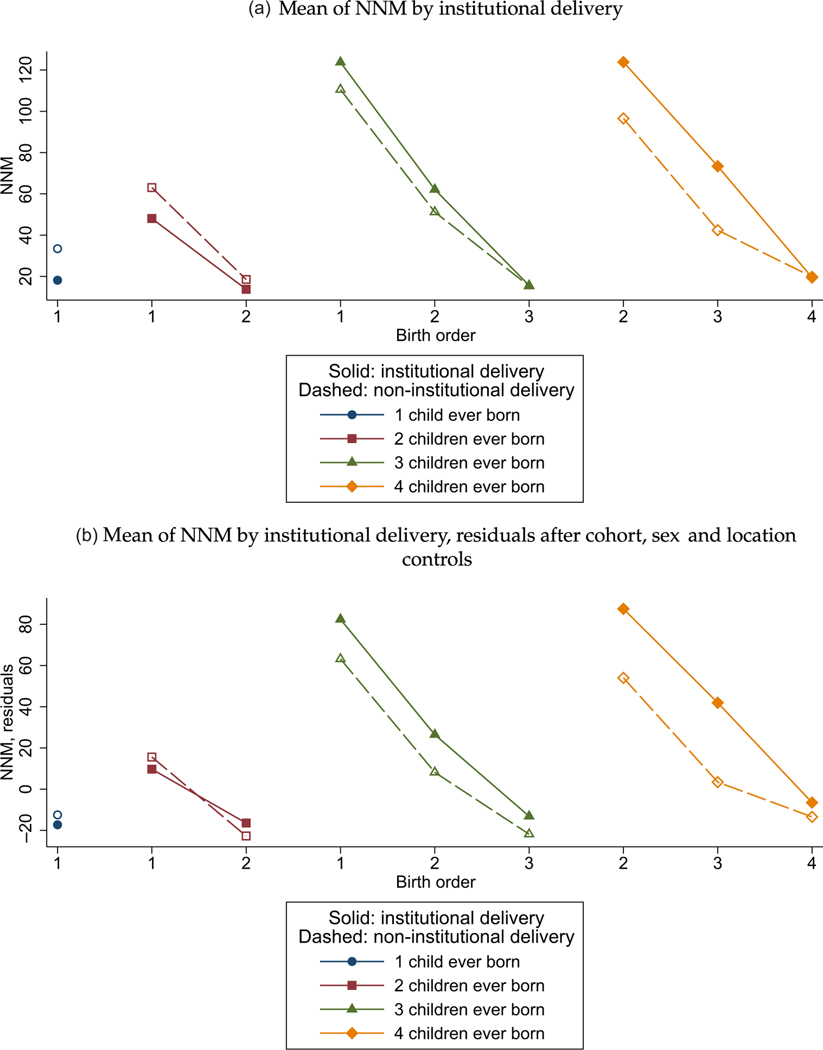
Institutional Delivery Does Not Explain India’s Birth-Order Pattern of NNM. *Notes:* The sample comprises India’s most recent DHS, from 2005–2006. In the DHS institutional delivery is only recorded for children under five years old, so only these ages are included in our sample. Controls are for a quadratic of birth cohort of the child and of the mother (both as CMC codes), child sex and whether the household is in a rural or urban location. Marker shapes indicate the number of children ever born to a mother by the time of the survey. For full regression results, see Online Appendix Table A.5.

Figure 3 shows that institutional delivery does not explain the effect of birth order. The pattern of neonatal mortality by birth order and sibsize is essentially identical among Indian children born in a health facility (filled markers) and among Indian children born in a home (open markers). Indeed, institutional delivery is not even consistently associated with reduced mortality. This result is consistent with evidence in the literature that rapidly increasing institutional delivery in India has not lead to improvements in health outcomes,^20^ because much of the care in health facilities is low quality (Chaudhury et al., 2006; Das et al., 2008), or is even rent seeking rather than health promoting (Coffey, 2014). Of course, our results are not intended to estimate any causal effect of institutional delivery—for example, they do not consider that women may give birth in hospitals because a pregnancy appears risky or a labour has been prolonged—they merely demonstrate that our birth-order results do not reflect medical care as a mechanism or as an omitted variable.

In the Online Appendix, Table A.5 shows these results in a regression framework. Controlling for an indicator for institutional delivery does not change the estimated effect of birth order on NNM. Moreover, it shows that, conditional on birth cohort, later-birth-order children are not more likely to have institutional deliveries. In Table A.5 results are presented with controls for sibsize indicators. The birth-order gradients in Table A.5 and Figure 3 must be interpreted with care because the sample of children for whom institutional delivery was collected differs from the main birth history sample: whereas the main result uses a mother’s complete birth history up until the time of the survey, data on institutional delivery are only collected for children under sixty months old. The consequences for a mother fixed effects regression are an example of what Miller et al. (2019) called ‘selection into identification’. The note to Table A.5 in the Online Appendix further discusses the importance of this sample restriction.

## 5. Mechanism: Maternal Nutrition and Anthropometry

Women in India gain social status as they progress through a childbearing career (Jeffery et al., 1988; Das Gupta, 1995). Because this process has consequences for food consumption and for work expectations, it has consequences for the net nutrition of mothers, and therefore also for the children they nurture during pregnancy and while breastfeeding (Palriwala, 1993; Das Gupta, 1995). Improvement in women’s social status occurs in part due to having children, in part due to increasing autonomy of the nuclear family from the joint family, sometimes in part due to death of the husband’s parents, and finally in part due to the passage of time and the social rank associated with age itself.

We have seen that the effect of birth order on NNM cannot be explained by medical care. We propose that our NNM results can be explained by India’s exceptionally poor maternal nutrition combined with weight gain and improvements in social status over her childbearing career.^21^ We show that young women in India are highly likely to be underweight, but the prevalence of underweight falls at a steep rate as women age and bear children. This is consistent with our main results: many babies in India would be born to undernourished mothers, and therefore would suffer from low birth weight. However, some babies—born later in their mothers’ childbearing careers—would have better outcomes, less different from those of children in the rest of the developing world because their mothers would be more nearly as likely to be underweight as mothers in the rest of the developing world.

This section provides a collage of evidence for our interpretation. Ideally, we would have data that would allow us to see how much of India’s NNM–birth-order patterns can be accounted for by controlling for a mother’s pre-pregnancy BMI and weight gain in pregnancy, for each of her pregnancies (Thomson and Billewicz, 1957; Siega-Riz et al., 1994; Abrams and Selvin, 1995; Yaktine and Rasmussen, 2009). Alternatively, we might control for a child’s birth weight, an important summary measure of her growth *in utero* and a predictor of her subsequent health (Behrman and Rosenzweig, 2004; Alderman and Behrman, 2006). Either of these strategies would require temporally precise anthropometric measurements from the time of the pregnancy or birth. Instead, the DHS measures the weight and height of adult women and some of their children only once, at the time of the survey. Therefore, we use cohort-tracking methods from the demography literature to document India’s pattern of maternal underweight and compare it to the rest of the developing world.

First, in Subsection 5.1 we discuss what can be learned from cross-sectional measurements in the DHSs. Next, Subsection 5.2 matches cohorts of adult women across successive cross-sectional DHS rounds to compare the mean consequences of cohort ageing for body mass in India to consequences of cohort ageing elsewhere. Then, in Subsection 5.3 we note that an alternative, longitudinal data set also shows a unique pattern of maternal nutrition in India. In Subsection 5.4 we study geographic heterogeneity beyond the India-other comparison, including by comparing states within India where maternal undernutrition is more and less common. Finally, in Subsection 5.5 we document that the effect of birth order on NNM in India is consistent with the correlation of the birth-order–NNM gradient in a DHS round with that DHS round’s age gradient of women’s undernutrition. We show that in DHS rounds where a woman’s age does not predict her chances of being undernourished, there is no protective effect of later birth on NNM.

### 5.1. Anthropometry in the DHSs Cross Sections

#### 5.1.1. The effect of parity progression on maternal anthropometry is difficult to study in the DHS cross section

The fact that DHS anthropometric data on the body sizes of mothers is collected cross sectionally presents two challenges for demonstrating that maternal nutrition is the mechanism behind India’s NNM–birth-order gradient. First, it is *incomplete*, in that there is not a measurement for each studied birth; rather mothers are measured only once however many children they have. Second, it is *mistimed*, in that we know the size of mothers at the time of the survey, when we would ideally like to know their sizes at the time of pregnancy and birth. Infant mortality data, which we have studied up to this point in the paper, have neither problem: in the DHS, mothers report all children ever born and the time of a child’s death.

Because a mother’s anthropometry in the DHS is only observed after her most recent birth, any cross-sectional correlation between BMI and parity at last birth would reflect a combination of the effects of (1) parity progression, meaning the longitudinal process that we wish to study, and (2) fertility selection, meaning any correlation between the number of children ever born to a mother and her anthropometry.^22^ Unlike in other developing countries on average, in India, these two effects have opposite signs and large magnitudes.

**Fig. 4.**
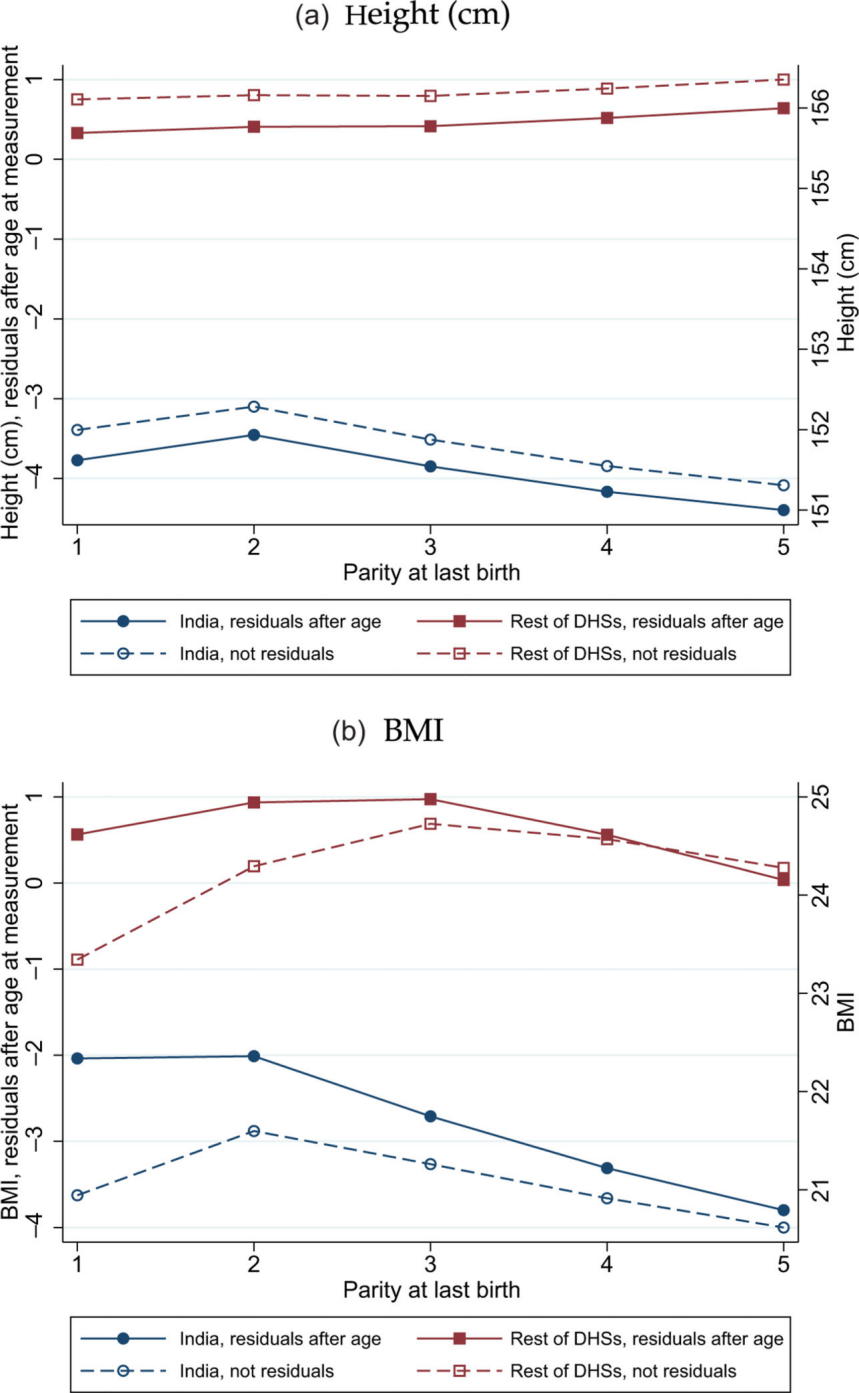
Differences in Fertility Selectivity between India and the Rest of the DHSs: Women’s Heights and Women’s BMIs, by Parity at Last Birth. *Notes:* Data taken from the women’s anthropometry sample described in Sections 2 and 5. For a similar figure using the alternative sub-Saharan Africa comparison sample, see Figure A.6 in the Online Appendix.

Figure 4 illustrates that high fertility is differently selective for a mother’s health in India and in the rest of the DHSs. It uses height and body mass index as outcomes, and shows that higher fertility tends to be correlated with anthropometric *advantage* in the rest of the DHSs, but with anthropometric *disadvantage* in India. The results for height are the same whether or not age is controlled for because adult height does not substantially change as a person ages. For BMI, the differences between India and the rest of the developing world are sharper once the woman’s age is controlled for (the solid lines and markers) because otherwise the shape of the relationship between BMI and parity reflects the combination of the selectivity of high fertility with the effect of progression through the life course.

The evidence of Figure 4 is consistent with the pattern we saw for NNM in Figure 1: the vertical distance between lines showed that, although within a sibsize later borns are advantaged, children born to larger sibsizes are substantially disadvantaged relative to children born to smaller sibsizes. Misinterpreting the correlations in Figure 4 simply as a longitudinal effect of parity progression would be misleading, similar to how it would be misleading to compare NNM between earlier-and later-birth-order children without accounting for sibsize.

We also note that Figure 4 shows large level differences in women’s health between India and the rest of the developing world: mothers in India are substantially shorter and have less body mass, on average, than mothers elsewhere. Although it is not shown in the figure, we note that average fertility is lower in India than in the other countries in these data.

#### 5.1.2. What can we learn from age patterns in the DHSs?

We have seen that it would be difficult to learn about the average pattern of maternal nutrition over a child-bearing career by studying the cross-sectional correlation of women’s BMI with parity progression in the DHSs. In this section, we study cross-sectional correlates of age: although comparing women of different ages in a cross section does not cleanly isolate a longitudinal process, it provides useful suggestive evidence. Because adult mortality is low at childbearing ages, age is not inherently selective in the way that parity is: almost every 20-year-old woman eventually becomes a 30- and a 40-year-old woman, but not every woman with one or two children eventually has three or four.

**Fig. 5.**
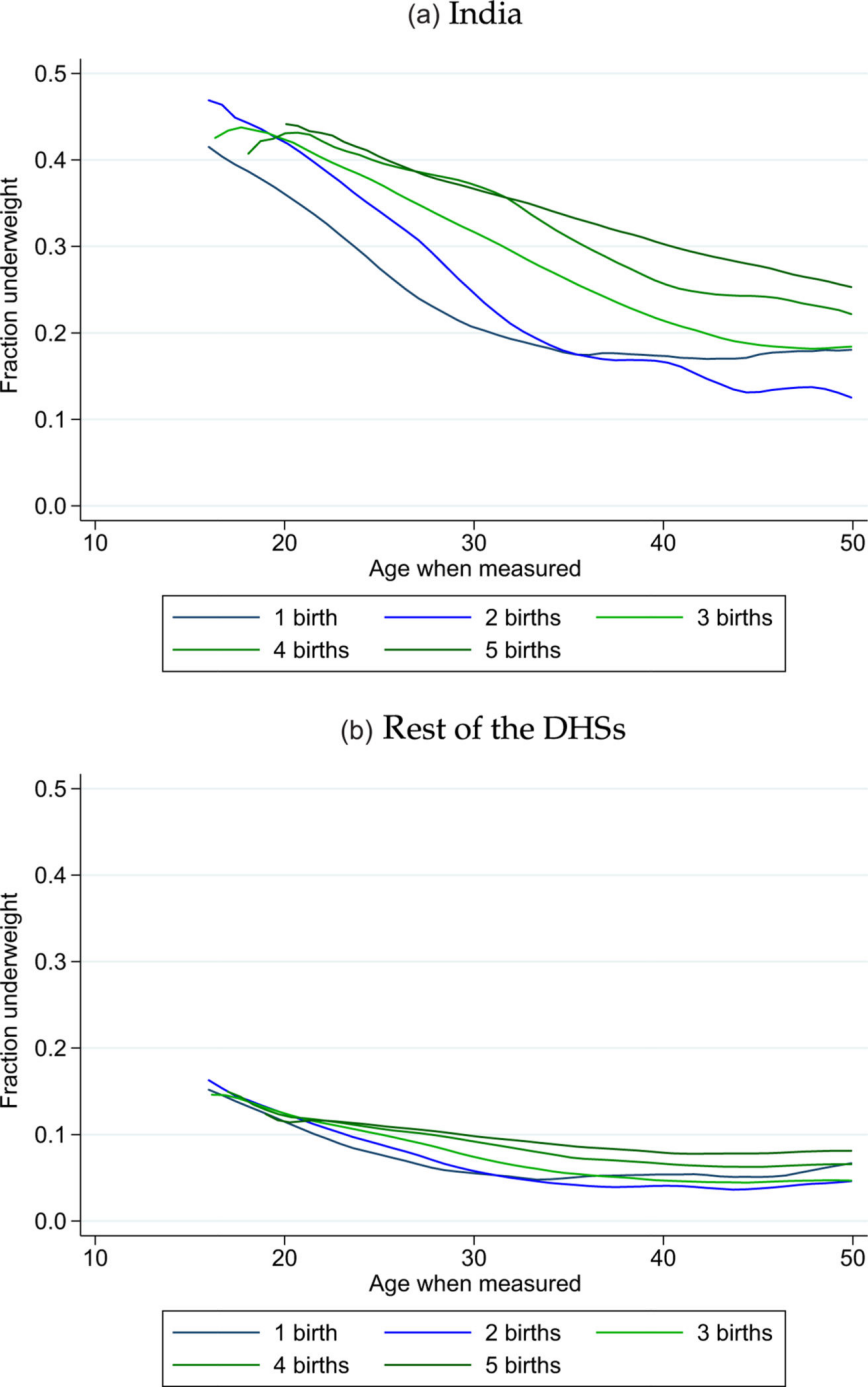
Women’s Underweight Is Predicted by Age in India, but Not in the Rest of the DHSs. *Notes:* Data taken from the women’s anthropometry sample, described in Sections 2 and 5. Vertical axes plot the fraction of women who are underweight, meaning that they have a body mass index below 18.5. Age, underweight and parity are all measured at the time of the DHS.

Figure 5 documents two facts about underweight (meaning a BMI below 18.5) among women in India, relative to the rest of the DHSs: (1) underweight is far more common in India, and (2) the cross-sectional age gradient of underweight is far steeper. That is, young women in India are especially likely to be underweight. Women at the end of their reproductive careers are less likely than younger women to be underweight, although still more likely than women in the rest of the developing world.^23^ These figures visually resemble the NNM–birth-order gradients shown in Figure 1.^24^

### 5.2. Evidence from Tracking Cohorts Across Repeated DHS Cross Sections

We have seen cross-sectional evidence that age is strongly associated with being underweight among mothers in India. For this pattern to account for the effect of birth order, however, it must reflect a longitudinal process that occurs as women progress through childbearing careers. Although DHSs are not individual-level panels, it is possible to track age cohorts through successive cross-sectional survey rounds. In other words, we can compute the average BMI among 20-year-old women in the 1998 Indian DHS, and compare this sample mean with the average BMI in the 2005 DHS, among women who *were* 20 years old at the time of the 1998 survey. Then, because adult mortality at these ages is low, and because the DHS is a representative survey of women at these ages, the average cohort-level longitudinal rate of change in mean BMI for that cohort is the difference between these averages divided by the length of the time interval.

In this section we report these cohort-level computations. We use data on all measured adult women from the women’s anthropometry sample described in Section 2, except that a woman can only be included if she is in a country that has had more than one DHS round that measures women’s anthropometry. Cohorts are matched across each successive pair of survey rounds within the same country. The DHS data have detailed information on age; so we are able to define cohorts by age in months at the time of the earlier survey round.

**Fig. 6.**
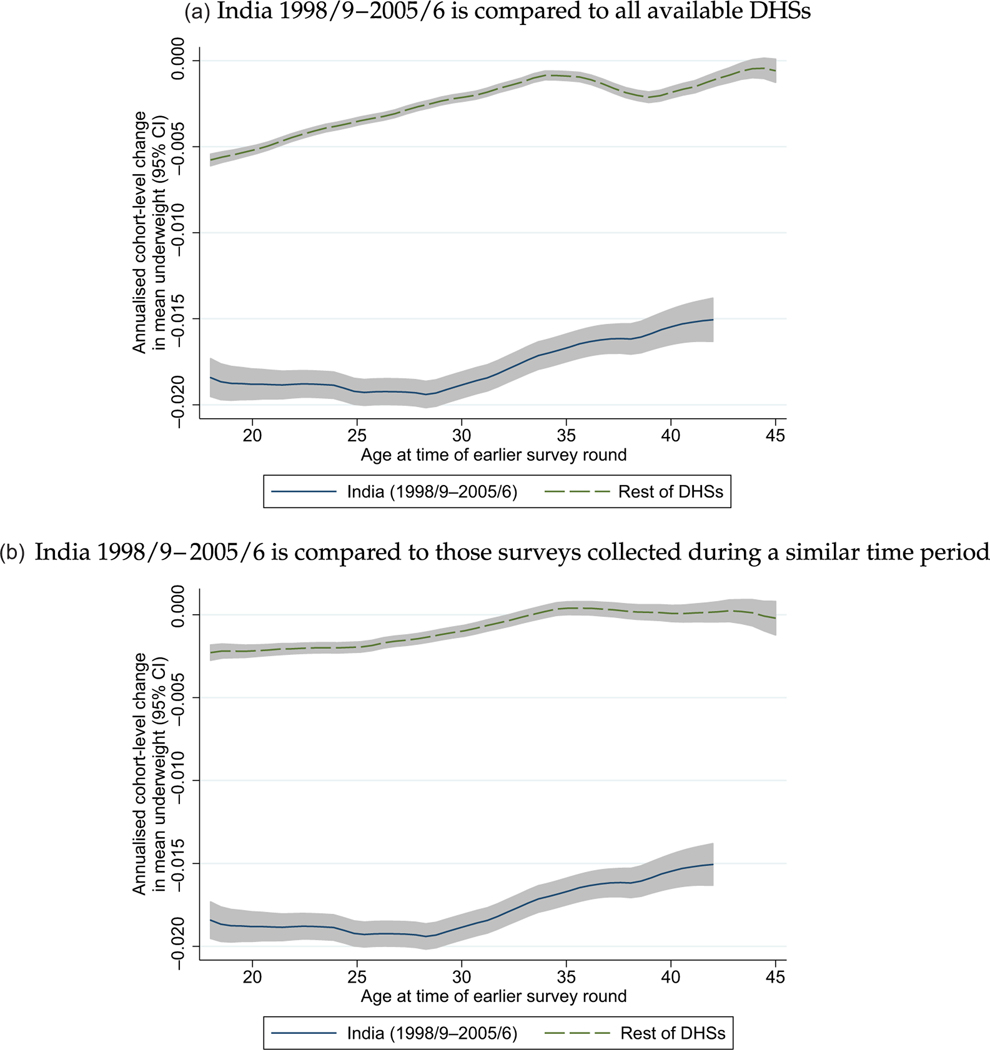
The Month-of-Birth Cohort-Level Rate of Change of Women’s Underweight Differs between India and the Rest of the DHSs. *Notes:* Panel (a) restricts the women’s anthropometry sample described in Sections 2 and 5 to those surveys for which there is more than one DHS round in the same country. Panel (b) restricts the women’s anthropometry sample to those DHSs for which the first of two surveys in the same country was within 2.5 years before or after the 1998/9 Indian DHS. In both panels, cohort mean changes are annualised by dividing by the time interval in months between DHS rounds.

Figure 6 presents the results of a computation of cohort rate of change in BMI. Each woman is matched to the mean property of the cohort of which she is a member from the immediately prior survey round. Therefore, the figure plots sample means of cohort velocity $v_{ct}$ for cohort c, over the time interval from survey round t−1 to round t, computed as

(2) $$v_{ct}=\frac{\bar{{\text{underweight}}_{ict}}-\bar{{\text{underweight}}_{ict-1}}}{\bar{m_{ict}}-\bar{m_{ict}-1}},$$

where i is an individual woman in a survey round, ‘underweight’ is an indicator for being underweight, m is the century-month code of the month of the interview historical time (so the denominator is time between surveys measured in months), $\bar{{\text{underweight}}_{ict}}$ is the average of underweight in survey round t. In panel (a), cohort-level changes that occurred between the 1998 and 2005 Indian DHSs are compared to all other available pairs of DHSs. In panel (b), changes between the two Indian DHSs are compared to changes in those places for which the first of two surveys in the same country was within 2.5 years before or after the 1998 Indian DHS.

It is clear in the figure that the longitudinal change in cohort anthropometry is different in India from the rest of the DHSs: underweight velocity is more negative in India, indicating that on average a larger fraction of the cohort is moving out of the underweight range each year in India than elsewhere. This is in part because more women in India are originally underweight to begin with. Figure A.8 in the Online Appendix replicates Figure 6 using BMI, rather than an indicator for underweight, as the dependent variable and finds similar results.

### 5.3. Anthropometry in Young Lives Longitudinal Data

Because the DHS is a cross section, one strategy is to turn to other data. In order to study longitudinal weight gain of individual mothers, this section draws upon a panel data source that tracked the anthropometry of mothers in developing countries over time. The Young Lives^25^ data longitudinally measured the weight of mothers of young children in four developing countries: India, Vietnam, Ethiopia, and Peru. The survey is principally designed to follow 12,000 children (3,000 at each country site) through childhood (Wilson et al., 2006). Data are not nationally representative; for example, the Indian data are from seven districts in the states of Andhra Pradesh and Telangana.

Although not reported in detail here, summary statistics from these results are qualitatively consistent with our nationally representative cohort estimates from the DHSs: women in India begin weighing the least among the four countries in 2009, and experience the largest longitudinal weight gain over the four years studied. Therefore, the panel data that exist are consistent with our life-course interpretation of the DHS cross section. Further details are available in Coffey and Spears (2019).

### 5.4. Geographic Heterogeneity in the Effect of Birth Order

The main analysis of this paper compares India with the rest of the developing world, and interprets the differences as due to India’s distinctive pattern of maternal undernutrition. However, other heterogeneity also correlates with maternal undernutrition, and can be used to assess our interpretation. In the Online Appendix, Figures A.10 and A.11 report two such investigations.

Within India, there exists a broad set of cultural differences and differences in maternal nutrition between northern and southern states. Figure A.10 focuses only on the Indian data, and compares the birth-order pattern in the north Indian states of Uttar Pradesh and Bihar (where 33% of women are underweight) with the birth-order pattern in the south Indian states of Goa, Kerala and Tamil Nadu (where women’s social status is higher and 19% of women are underweight). At all birth orders, the effect on NNM of a later-order birth, relative to a first birth, is more negative in the northern states than in the southern states. Although the sample is much smaller here than in our main international sample, this interaction is statistically significant for the comparison of second with first births; unlike in the rest of the DHSs, these two birth orders are a majority of births in India, because India has lower average fertility than the rest of the DHSs.

Across the developing world, some localities outside of India have high rates of maternal undernutrition; some localities within India have relatively low undernutrition. Figure A.11 uses the main DHS sample, and substitutes an interaction between birth order and the local prevalence of underweight among adult women, instead of an interaction between birth order and India. Consistent with our interpretation that maternal nutrition drives our main results, the larger the fraction of adult women in a locality (PSU) who are underweight, the more negative the effect of later birth order on BMI. This interaction is statistically significant at all birth orders. Because underweight is much more common among adult women in India than in the rest of the DHSs, this interaction projects a more negative effect of later birth on NNM in India than in the rest of the DHSs—which matches what we find in our main results.

### 5.5. Connecting Maternal Underweight with the Effect of Birth Order

**Fig. 7.**
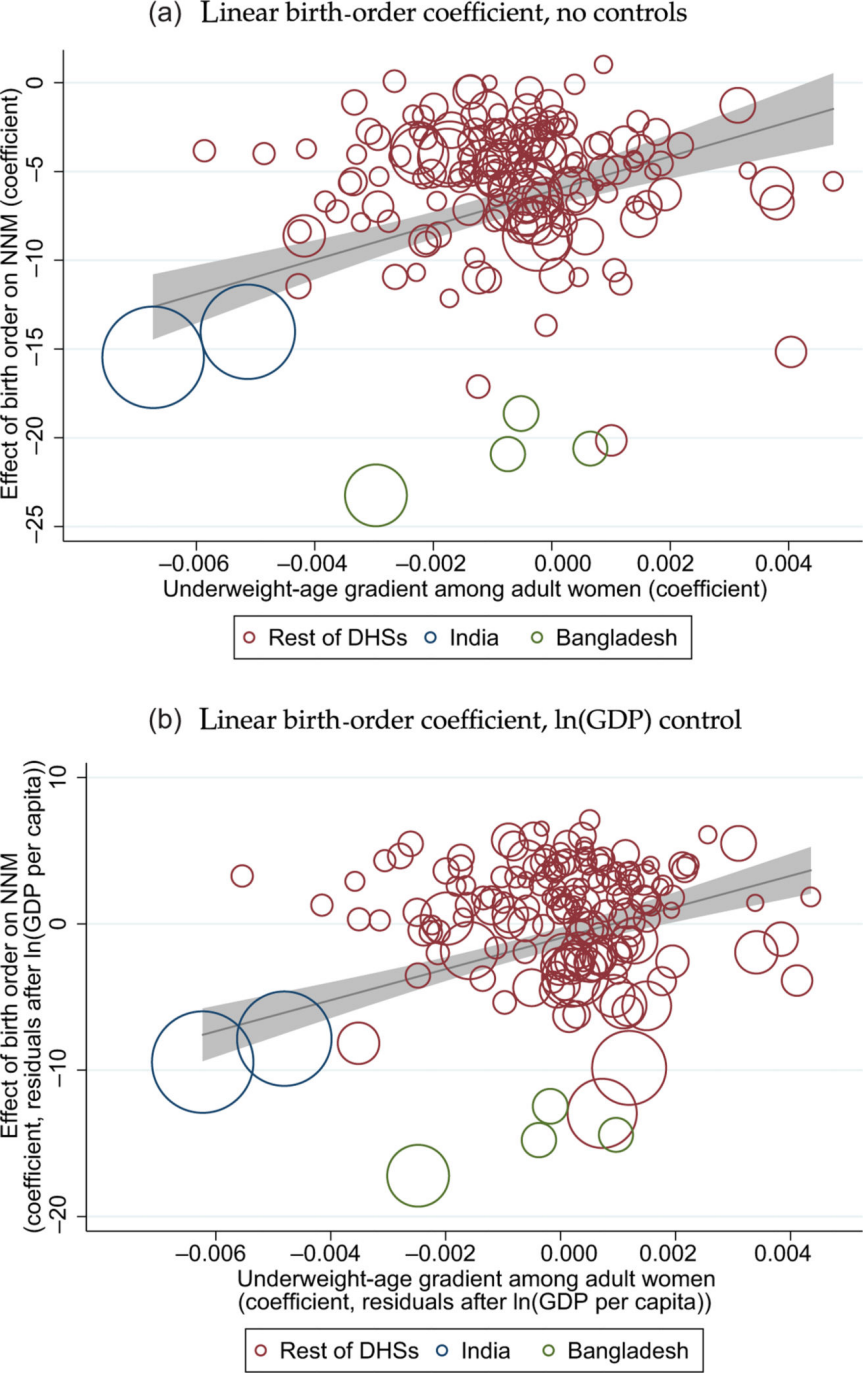
Across DHS Rounds, a Larger Negative Effect of Birth Order on NNM is Associated with a Steeper Negative Gradient between Age and Underweight among Adult Women. *Notes:* The sample comprises DHS rounds used to construct the main DHS sample of births discussed in Section 2. In both panels, each point plots regression results from two separate regressions, each estimated for one DHS round at a time. The vertical axes plot the coefficient on birth order linearly predicting NNM, in a regression with mother fixed effects. The horizontal axis plots the coefficient on adult women’s age in years linearly predicting an indicator for being underweight. The results in panel (b) additionally control (by residualising the variables in the horizontal and vertical axes in two separate regressions) for a DHS-round-level mean of GDP per capita; for more detail, see Subsection 5.5.

Figure 7 presents results at the DHS-round level that integrate the mortality results with the results on women’s anthropometry. In particular, this figure shows that India’s exceptional effect of birth order is consistent with its exceptional age profile of maternal underweight. Each point in the figure reflects two separately estimated regressions, both estimated using data from one of the survey rounds in the main DHS sample of births. The vertical axes plot the coefficient on birth order linearly predicting NNM, in a regression with mother fixed effects.^26^ The horizontal axis plots the coefficient on adult women’s age in years linearly predicting an indicator for being underweight.

Because this scatter plot is an international comparison of country years at different stages of development, the reader might wonder whether the pattern in Figure 7 merely reflects socioeconomic development. Therefore, panel (b) adds to panel (a) a control for GDP per capita. The same DHS-round-level coefficients are used in panel (b) as in panel (a), but now the coefficients on the horizontal and vertical axes are residualised after regressing the set of coefficients from each axis on GDP per capita.^27^ The control for economic development does not change the conclusion: panel (b) resembles panel (a). This is consistent with our basic observation that India’s early-life health differs from other countries at similar levels of economic development, because its patterns of maternal undernutrition do, too.

The fact that the association between these two gradients is positive means that the places and times in which the age-underweight gradient among adult women is more negative are those in which being later born confers more of a neonatal survival advantage. The fact that the trend passes through the origin means that in DHS rounds where there is no age-underweight gradient among mothers, there is also no effect of birth order on NNM. Finally, we note that the large blue circles, which represent India, are at the far left of the graph, meaning that both of the relationships we study are exceptionally negative compared to other countries. However, the data points for India are also consistent with the international trend, which is shown in the figure.^28^ Of course, these computations should be interpreted with the same care as is required for interpreting Figure 5: the horizontal axis of these graphs reflects a regression on age in a cross section, not longitudinal parity progression. We therefore emphasise merely that, across independently computed regressions on separate samples, India’s protective effect of later birth on NNM is linked with its age pattern of maternal nutrition; one does not appear without the other.

Figure A.12 in the Online Appendix verifies that these results are similar if, instead of looking at the association of the magnitude of regression coefficients, we instead plot the association of test statistics for the coefficients of interest from the regressions described above. In Figure A.12(a) and (b) we plot the t-statistics for the same linear regression coefficients plotted in Figure 7. t-statistics have the advantage over regression coefficients of not giving importance to noisy estimates. Panels (c) and (d) replace the vertical axis with F-statistics (multiplied by the sign of the linear coefficient) from a regression of NNM on birth-order indicators, rather than birth order entered linearly.

## 6. Magnitude: How Different would NNM in India be without the Effect of Birth Order?

How large of a total impact on India’s aggregate NNM does its unique effect of birth order amount to? One version of this question is to ask: by how much would NNM in India be reduced if it matched the rest of the developing world’s birth-order effect, keeping the effect of sibsize unchanged? This is a counterfactual question, for which we can use our estimates to compute a projection in the spirit of a demographic reweighting that weights subsample-specific changes by their frequency in the population. It is also, we emphasise, a *hypothetical* accounting question: although this is an informative way of scaling our effect estimates, no actual policy could change the correlation between birth order and mortality without also changing, for example, the correlation between sibsize and mortality.

We ask by how much India’s average NNM would change if each combination of birth order and sibsize switched from the NNM it actually experiences to the NNM it would counterfactually experience if its disadvantage relative to the NNM of the last born of its sibsize matched the earlier-born disadvantage experienced by that birth order and sibsize combination in the rest of the developing world. This computation leaves mortality among last births unchanged. Because, on average, being earlier born carries an larger average NNM penalty in India, this counterfactual change would reduce NNM in India.

To construct a projected NNM, we compute a counterfactual change in India’s NNM with the following weighted sum, where b continues to index birth order and j indexes sibsizes, both running from 1 to a category for 6 or greater:

(3) $$\text{counterfactual}\;\text{Δ}\;{\text{NNM}}_{\text{India}}=\frac{\sum_{1\leq j\leq 6+}\sum_{1\leq b\leq j}w_{bj}d_{bj}}{\sum_{1\leq j\leq 6+}\sum_{1\leq b\leq j}w_{bj}}.$$

Here $w_{bj}$ is the weight of the combination of birth order b and sibsize j in the Indian sample, and $d_{bj}$ is the difference that our counterfactual change in mortality rates would make to that partition of the births. Our computations of $\text{Δ}\;{\text{NNM}}_{\text{India}}$ differ in three ways.

1. *Sample*: we use either the full sample (as in Figures 1 and 2) or the restricted sample intended to focus on completed fertility (as in Figures A.1 and A.3).
2. *Weights* $w_{bj}$: we weight our sample to reflect the observed distribution of births in India’s 2005–2006 DHS, its most recent. Our two approaches to weights $w_{bj}$ either sum the DHS sampling weights^29^ across all births of birth order b and sibsize j, or alternatively ignore the sampling weights and set $w_{bj}$ equal to the count of births.
3. *Differences* $d_{bj}$: our first approach to constructing a counterfactual difference is to use the summary NNM statistics plotted in Figures 1 and A.1. We ask how NNM for birth order b in sibsize j compares to birth order for the last borns j of sibsize j, and how this difference in India compares with that elsewhere:
(4) $$d_{bj}=\left({\bar{\text{NNM}}}_{bj}^{\text{India}}-{\bar{\text{NNM}}}_{jj}^{\text{India}}\right)-\left({\bar{\text{NNM}}}_{bj}^{\text{rest of DHS}}-{\bar{\text{NNM}}}_{jj}^{\text{rest of DHS}}\right).$$
  Our second approach uses the regression coefficients on the interaction between India and birth order from (1), plotted in Figure 2:
(5) $$d_{bj}={\hat{\beta }}_{1}^{b}-{\hat{\beta }}_{1}^{j},$$
  with ${\hat{\beta }}_{1}^{1}$, the omitted category in the regression, set to zero.

The regression approach has the advantage of controlling for the full set of regression controls, including mother fixed effects (FEs), sex and cohort, but has the disadvantage of restricting the birth-order gradient to be constant across sibsizes. Note that either definition of $d_{bj}$ sets $d_{jj}=0$ for all last borns of a sibsize. This is because the counterfactual exercise is to match the rest of the world’s NNM disadvantage of earlier-born siblings relative to last-born siblings, leaving unchanged fertility selectivity, as reflected in the average NNM of the last born of each sibsize.

Table 1 presents the results. NNM in India, it is projected, would be lower by eight to ten deaths per 1,000 births if it matched the rest of the world’s birth-order gradient. This is about the quantitative size of the gap between India’s actual NNM and what is predicted by India’s GDP per capita (Coffey and Hathi, 2016). Results are approximately quantitatively similar across the eight combinations of counterfactual strategy. To be sure, these comparisons must be considered with care: there is no reason to assume that the effect of birth order in India *would* precisely match the rest of the developing world in the absence of the particular social forces we study.^30^

A difference of nine neonatal deaths per 1,000 births in India would be large. In 2015, 3.5 billion people lived in a country where the *total* NNM was less than nine per 1,000. These countries include China, Brazil, Russia, Mexico and Thailand. Such a reduction in India would eliminate 8% of all neonatal deaths that occur each year, worldwide.^31^ Because there are about 13 million neonatal deaths each five-year period, this would account for an annual difference of a little over 200,000 infant deaths, if there were no countervailing increase in post-neonatal mortality.^32^

## 7. Conclusion

In this paper we examine birth order and early-life mortality in developing countries, using birth histories that allow us to separate the effects of birth order from correlates of sibsize and maternal and child cohort of birth. We find a unique later-born neonatal mortality advantage in India that is much steeper than in the rest of the developing world. This later-born advantage contrasts with well-known findings from the developed world that later-birth-order children are disadvantaged in human capital (Black et al., 2005).

The effect of birth order on NNM in India is unusual, but it is explicable: it is consistent with the level and pattern of maternal undernutrition and social status among women in India (Jeffery et al., 1988). We show that high maternal undernutrition is concentrated among young women early in their childbearing careers. Steep differences in NNM by birth order are consistent with steep improvements in maternal nutrition that we document across childbearing careers. Moreover, comparing effect estimates across DHS rounds, we show that India’s birth-order effect on NNM is what the international trend predicts, given this pattern of maternal weight.

Our investigations of health outcomes have potential methodological implications as well as substantive implications: within India, the correlation of health outcomes with sibsize and with birth order have opposite signs. Researchers using cross-sectional data (such as the widely used DHSs) to study effects of birth order or parity progression on anthropometric outcomes (such as mothers’ BMI or child height) may obtain biased estimates unless their empirical strategy can separate birth order or parity progression from sibsize and fertility (Spears et al., 2019).

Our estimates are of such quantitative magnitude as to constitute important facts about the overall composition of infant death in a world population in which more than a quarter of neonatal mortality occurs in India. Moreover, these findings demonstrate the continued relevance of woman’s status, household structures and demographic relationships to health outcomes in the developing world. Because early-life health is of enduring importance for economic and later-life health outcomes, the welfare consequences of these patterns for the Indian population are surely very large. They recommend policies to reduce maternal undernutrition; they also caution that discrimination against young women could perhaps prevent nutritional inputs intended for pregnant women from benefiting them and from promoting their babies’ survival.

## Supplementary Material

Supp Material

## Figures and Tables

**Table 1.** Counterfactual Projected Decrease in Indian NNM, if it were to Match the Birth-Order Gradient in the Rest of the Developing World.

Method	Note	Equal weights	Sample weights
Mean differences	Full sample	8.1	8.6
Mean differences	Restricted sample	9.8	10.0
Regression	Mother FEs & controls	10.6	10.9
Regression	Sibsize controls only	11.1	11.2

*Notes:* The table presents eight alternative estimates of the counterfactual decrease in India’s NNM from matching the rest of the world’s NNM gradient in birth order, while holding constant the average NNM among the last-born children of each sibsize. The purpose is to summarise the magnitude of the effect that we document (not to evaluate any actual policy proposal). For full details of the computation, see Section 6. The second row uses the restricted sample (described in Section 2), which excludes births to mothers whose last birth was within five years of the survey. The estimates in the third and fourth rows use the main DHS sample of births.

## Footnotes

1 Sources and computations for these motivating demographic facts are fully detailed in Section A of the Online Appendix.

2 We use the abbreviation NNM for the neonatal mortality rate: neonatal deaths per 1,000 live births.

3 Other studies from developed countries that document later-born disadvantages include Conley and Glauber (2006) and Price (2008). A smaller literature has studied developing countries. Studying rural India, Behrman (1988) found an earlier-born advantage in intrahousehold allocation—the opposite of the direction of our result. Studying educational outcomes in households in Ecuador where all children of the mother are alive, De Haan et al. (2014) found a later-born advantage in poorer households and an earlier-born advantage in richer households.

4 Some recent studies also note later-born advantages in early-life health and related variables in Scandinavian data (Lundberg and Svaleryd, 2017; Brenøe and Molitor, 2018); see also Modin (2002).

5 Chen et al. (2016) summarised: ‘in the unrestricted samples, the US excess mortality ranges from 1.4 to 3.6 deaths per 1000.’ They studied Austria, Belgium, Finland, the UK and the United States, which UN World Population Prospects data compute have slightly more than five million births per year combined, compared with about 26 million in India. Chen et al. (2016) cited Viscusi and Aldy (2003) as reporting that the value of a statistical life in the United States is $7 million; applying the $1 million for India reported in that review article would suggest that the approximately 200,000 annual excess neonatal deaths that we compute (see Section 6) are worth about $200 billion, assuming that eliminating these deaths would not increase post-neonatal mortality. Replicating the Chen et al. (2016) calculation suggests that India should be willing to spend about $7,500 per live birth to eliminate its effect of birth order on NNM. Although these computations are necessarily approximate, what is clear is that the effects in question are large.

6 The Annual Health Survey 2011 estimated an NNM of 50 for Uttar Pradesh; in 2015 the globally greatest country-level NNM reported in the World Bank World Development Indicators was 49.

7 Consider the Million Death Study (Million Death Study Collaborators, 2010), in which the following was noted of India in 2005: ‘three causes accounted for 78% (0.79 million of 1.01 million) of all neonatal deaths: prematurity and low birthweight (0.33 million, 99% CI 0.31 million to 0.35 million), neonatal infections (0.27 million, 0.25 million to 0.29 million), and birth asphyxia and birth trauma (0.19 million, 0.18 million to 0.21 million).’ Despite emphasising size at birth as the leading cause, it does not emphasise policies to improve maternal nutrition: ‘Expanded neonatal and intrapartum care, case management of diarrhoea and pneumonia, and addition of new vaccines to immunisation programmes could substantially reduce child deaths in India.’

8 See also Subsection 4.3. Institutional delivery figures are from the 2015–2016 National Family Health Survey fact sheet. Thirty-four percentage points of this 40 percentage point difference are from births at public, rather than private, facilities. NNM is from India’s Sample Registration System.

9 Coffey (2015b) first noted the cross-sectional age gradient of undernutrition among women in India in the demo-graphic literature, but did not study longitudinal weight gain or children’s mortality outcomes.

10 By design, many DHS rounds do not measure the height and weight of mothers. We exclude these surveys because these variables are critical to our analysis in Section 5.

11 This approach is common in the demography literature, although it is subject to both exclusion errors (women who have completed their last birth more recently than five years ago) and inclusion errors (women whose last birth was over five years ago, but who will give birth again) (see Bhalotra and Van Soest, 2008). In our sample, 82% of second–sixth borns were born after an interval of less than five years from the prior reported birth. As we will show, our results are qualitatively and quantitatively robust to using this restricted sample instead of our main sample.

12 Comparing India with sub-Saharan Africa is an enduring focus in the literature (e.g., Drèze and Sen, 2013; Nisbett, 2017). Our alternative comparison sample is the same sample of African DHS rounds used to study the India–Africa height gap by Jayachandran and Pande (2017) and Spears (2018). Coffey (2015b) compared maternal nutrition in India and Africa using a slightly different sample of African survey rounds.

13 Where the DHS measures women’s anthropometry, it either measures all women aged 15–49 in the full sample of households, or all of the women aged 15–49 in a randomly selected subsample of households. In both types of surveys, DHS data on women’s anthropometry are nationally representative of women of childbearing age in the country year in which the survey was done.

14 In a robustness check that is available in the replication files, we instead use logistic regression, which effectively estimates the *ratio* of the birth-order effect in India to that elsewhere. We find the same qualitative pattern, which is an important verification of the fact that our results are not driven by the zero lower bound on mortality. We thank Irma Elo and Michel Guillot for this suggestion.

15 A century-month code (CMC) is a month-resolution measure of historical time. It is the month since January 1900, which is coded as 1. Therefore, April 2017 is 117 × 12 + 4 = 1,408.

16 Using DHS data, Jayachandran and Pande (2017) found that higher birth-order children in India are shorter than lower birth-order children in India, on average, and that this gradient between birth order and average height is steeper in India than in sub-Saharan Africa. Investigations into child height and family structure in India, described in Spears et al. (2019), suggest that this result is sensitive to specification choice: it reverses when controls for sibsize are introduced. That is, at the same sibsize, later-born Indian children are taller than earlier-born Indian children. This is because sibsize is mechanically correlated with birth order, especially in DHS data that only observes height for the youngest children. This finding is consistent with Figure 4 of this paper, which finds that larger sibsize (or higher mother’s fertility) is a marker of disadvantage in India but not in the rest of the DHSs.

17 For example, sibsizes of three could include, among other possibilities, (boy, then girl, then boy) or (girl, then boy, then boy).

18 This is because mothers whose earlier-born children are girls are more likely to go on to have more children.

19 For example, a mother who experiences a neonatal death is 18.8 percentage points more likely to go onto have a subsequent birth in the next 36 months in Africa and 18.9 percentage points more likely in India. This small difference is not statistically significant, and the sign of the India–Africa difference is different for births within the next 24 months. Comparing our effect sizes with these results, which are available in detail in the replication files (as well as considering the similarity of our results when controlling for sibsize, sibsize structures or mother fixed effects), suggests that this issue is not a problem in our case. We thank Andrew Foster for suggesting this analysis.

20 Lim et al. (2010) studied the recent rapid expansion of institutional delivery in India as part of a conditional cash transfer program. Although institutional delivery is correlated with neonatal survival in the sample that they studied, they showed that, when a difference-in-differences empirical strategy that considers the extent of within-district implementation is used to identify a causal effect, there is no apparent effect on NNM (Online Appendix Table 3).

21 We cannot separate social status as a root cause from other causes of a steep trajectory out of underweight by parity progression in India: it is consistent with our findings and interpretation, for example, that low-status young mothers in India are very likely to begin childbearing careers while underweight, and that the biological processes of pregnancy cause them to gain weight that they otherwise would not gain and do not fully lose after giving birth.

22 Parity could also be correlated with age, cohort, socioeconomic status or education, all of which could be correlated with anthropometry.

23 Coffey (2015b) noted that working-age adult men in India are also more likely to be underweight than men in the rest of the developing world. The fact that high-status men and women have low body mass in India is consistent with evidence that social status is not the only determinant of poor nutrition in India: shared contextual causes also matter, such as disease externalities from open defecation (see Duh and Spears, 2017).

24 Figure 5 focuses on one cut point in the BMI distribution: the division between being underweight or not. In the Online Appendix, Figure A.7 extends this analysis by examining the cross-sectional association between adult women’s ages and the linear probability of being above a range of BMI scores. The figure shows that in India’s cross section, age predicts whether a woman has *normal, rather than low, BMI*, while in the rest of the DHSs age predicts whether a woman has *high, rather than normal, BMI*. See Online Appendix B for more details.

25 Data are available online at www.younglives.org.uk.

26 This is different from (1) in which birth order is entered as dummy variables. We use a linear term for birth order here in order to be able to plot a single parameter estimate for each DHS round.

27 A DHS-round-level GDP per capita variable is constructed by: (1) matching GDP per capita in the year of birth (from the Penn World Tables) to each birth in the main DHS sample of births that is used as an observation to produce the coefficient on the vertical axis, (2) taking the sample mean of these matched per capita GDPs and (3) taking the log of the mean.

28 Figure 7 also highlights Bangladesh, India’s South Asian neighbour and a notable case in need of further study, in which there is a relatively little underweight-age gradient but a relatively large birth-order gradient.

29 This is our only use of sampling weights in this paper, as we are intending to produce a summary statistic comparable to the published population-level demographic rates released in the DHS manual (Solon et al., 2015).

30 Note that, because mother fixed effects absorb household and village properties that are constant across siblings, this specification controls for any fixed properties of the mother such as her education, household or neighbours’ religion (Geruso and Spears, 2018), local sanitation (Coffey and Spears, 2017), or the mother’s birth order (Vogl, 2013) or that of of her husband.

31 Twenty-seven percent of neonatal deaths are in India. A nine per 1,000 reduction would reduce NNM from 31.7 to 22.7. Therefore, the percentage of neonatal deaths, compared to the actual number, would be (22.7/31.7)×27+73≈92.

32 Recall from Figure 2 that there is no offsetting decrease in PNM in birth order: the effect on IMR is almost the same as the effect on NNM. Online Appendix Figure A.5 verifies this further for survival up to 24 months.
