# Supplementary material for: NEONATAL DEATH IN INDIA: BIRTH ORDER IN A CONTEXT OF MATERNAL UNDERNUTRITION*
Source: Econ J (London). Author manuscript; Available in PMC 2024 Jul 12. (PMC11244657; doi:10.1093/ej/ueab028)
Supplement: Supp Material [file NIHMS1908736-supplement-Supp_Material.pdf]

A Introductory demographic summary statistics

- "Twenty-seven percent of neonatal deaths now occur in India."
  - Births per five-year period in thousands, from UN World Population Prospects (2010-2015): World: 699,214; India: 129,729. Thus 19% of births each year are in India.
  - Neonatal mortality rates (2015), from World Bank World Development Indicators (WDI), expressed per 1,000: World: 19.2; India 28.
  - These imply 3,632 neonatal deaths per five-years in India and 13,425 globally, both in thousands.
- Decline in IMR: World Bank WDI show 121.9 in 1960 to 31.7 in 2015, both per 1,000 births.
- "Over three-fifths of infant deaths are neonatal deaths: deaths in the first month of life.": World Bank WDI NNM in 2015 is 19.2 for the world, compared with 31.7 for IMR. 19.2 $\div$ 31.7 is 61%.

B How age predicts alternative BMI cutpoints

Figure 5 focuses on one cut-point in the BMI distribution: the division between being underweight or not. In the Supplementary Appendix, Figure A.7 extends this analysis by examining the cross-sectional association between adult women's ages and the linear probability of being above a range of BMI scores. The figure shows that in India's cross-section, age predicts whether a woman has *normal, rather than low, BMI*, while in the rest of the DHS age predicts whether a woman has *high, rather than normal, BMI*. A low BMI is one that is less than 18.5, a normal BMI is one that is between 18.5 and 25, and a high BMI is one that is greater than 25. For each BMI cutpoint, c, in half-point increments, for each sample s (India or the rest of the DHS), the figure plots the coefficients $\beta^s_{1,c}$ from the following linear probability model, with and without controls for children ever born:

$$\mathbf{1}\left[BMI_{ms} > c\right] = \beta^s_0 + \beta^s_{1,c}\text{age}_{ms} + \theta\text{children ever born}_{ms} + \varepsilon_{ms} \quad (6)$$

Thus, figure A.7 plots regression coefficients from separate regression estimations. All of the coefficients are positive because older adults tend to weigh more than younger adults in populations worldwide. The coefficients are larger for Indian women than for the rest of the DHS, meaning that age is especially predictive of BMI, and are especially large around the low BMI cutpoints, which is where maternal undernutrition poses a threat to

children. In other words, in India, the distribution of BMI for older women is different from the distribution for younger women around the *underweight* side of the distribution; in the rest of the developing world, age is associated with a shift of the distribution through normal BMIs to overweight. Controlling for the number children ever born (results shown with dashed lines) makes the age-BMI gradients more steeply positive because age, parity progression, and BMI are all positively correlated with one another, but higher-fertility women weigh less, on average, because they are poorer.

Figure A.1: Early-life mortality by birth order and sibsize, restricted sample

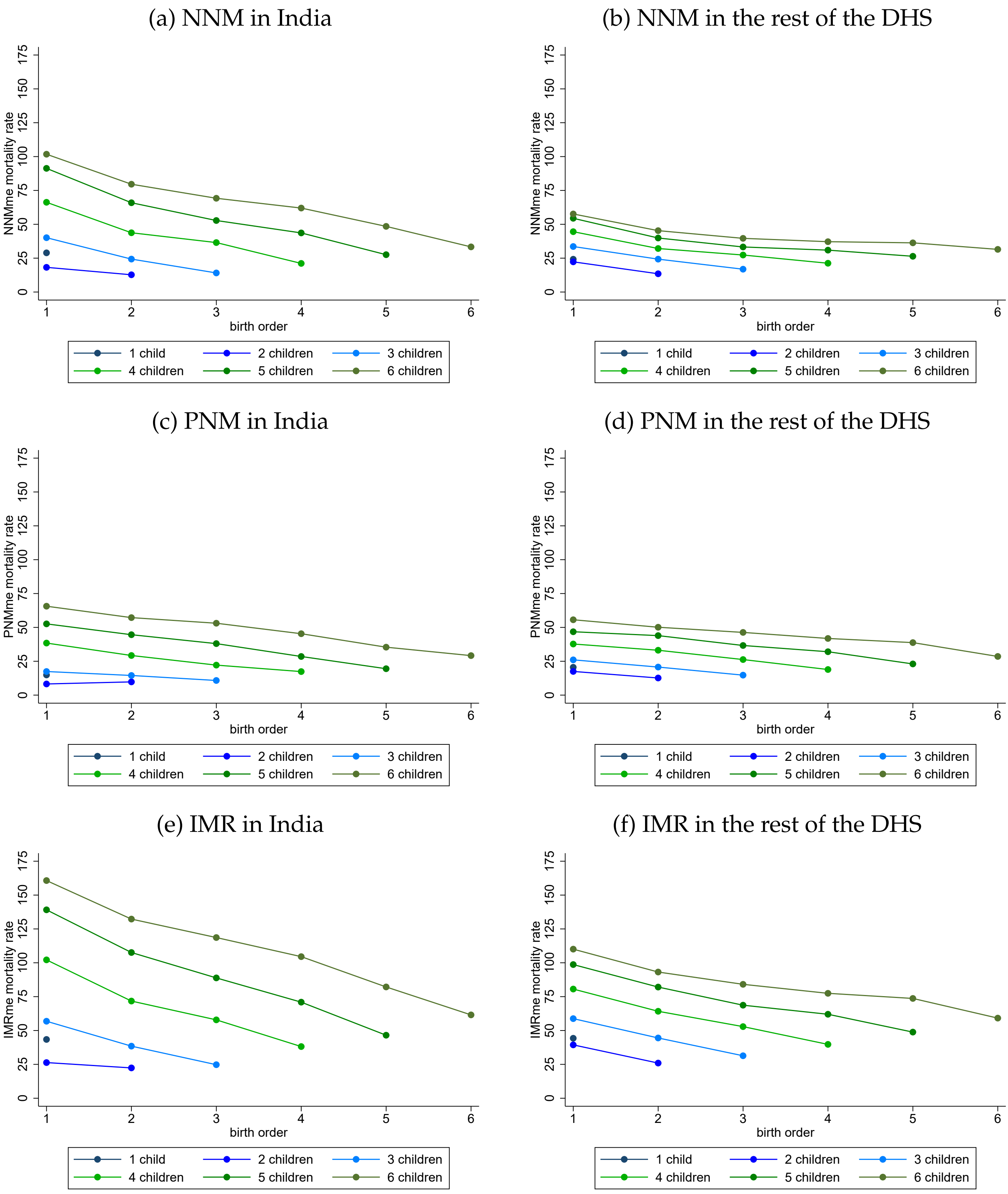

Restricted sample: Starting from the main DHS sample of births described in section 3, this sample excludes all births to mothers who have had a birth in the past five years to avoid confounding by incomplete fertility. Mortality rates are scaled to per 1,000.

Figure A.2: NNM by birth order and sibsize, replication with African sample

(a) NNM in India, full

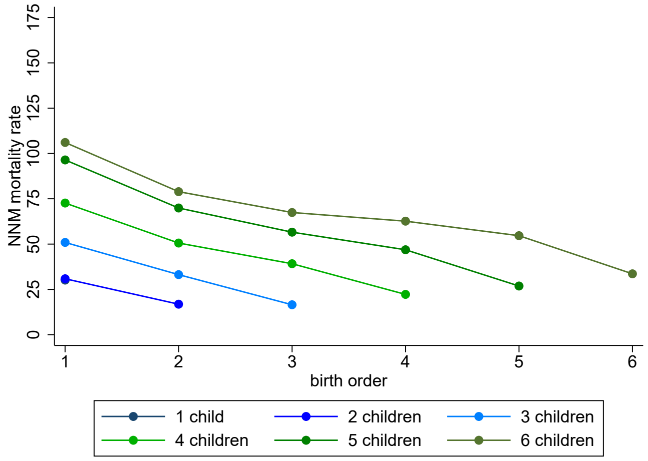

(b) NNM in India, restricted

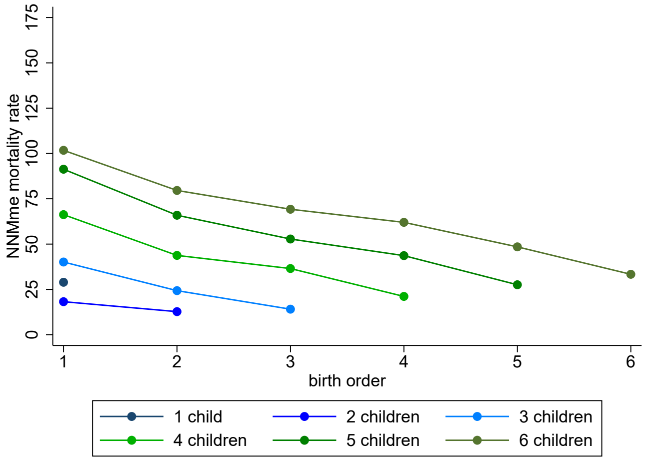

(c) NNM in the rest of the DHS, full

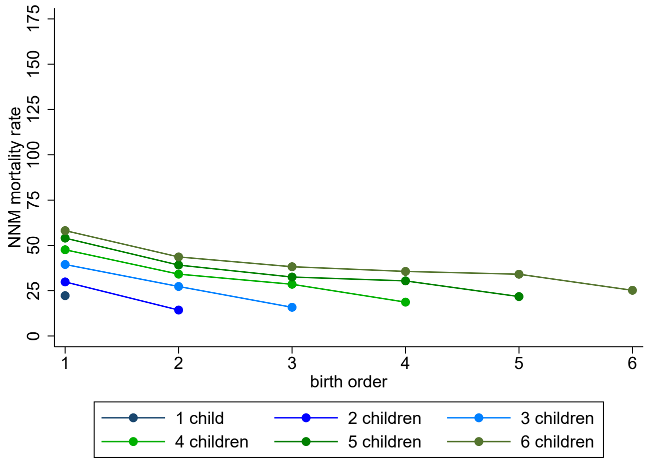

(d) NNM in the rest of the DHS, restricted

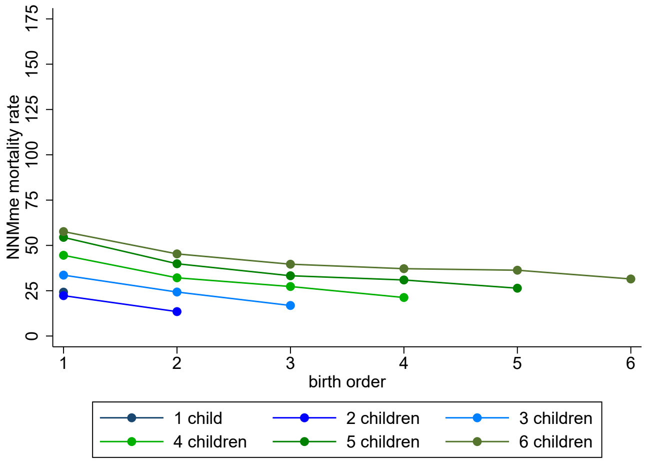

(e) NNM in Africa, full

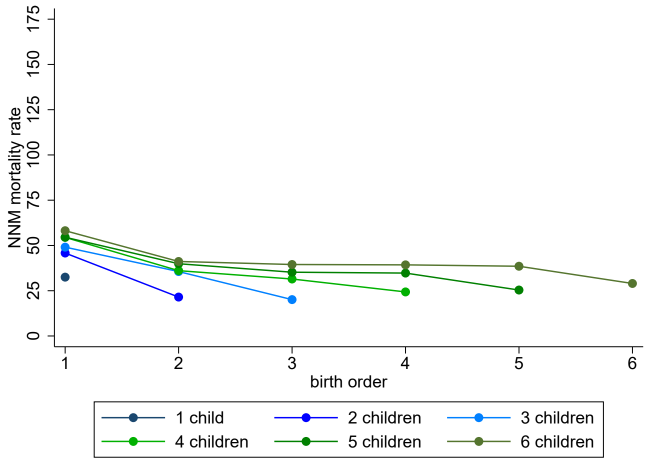

(f) NNM in Africa, restricted

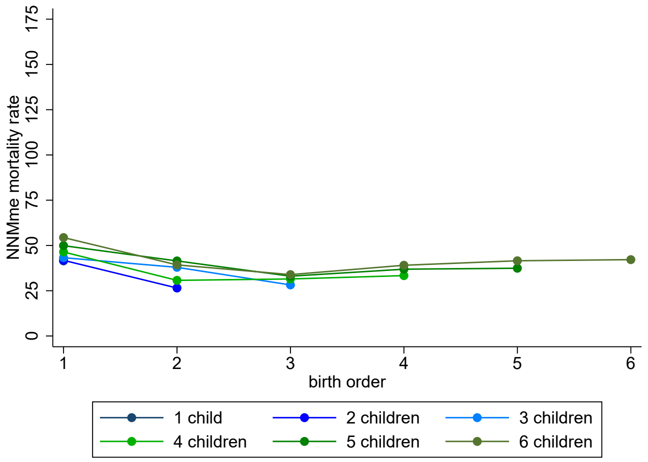

Restricted sample: Starting from the main DHS sample of births described in section 3, this sample excludes all births to mothers who have had a birth in the past five years to avoid confounding by incomplete fertility. African sample: This sample includes a set of DHS survey rounds used by Jayachandran and Pande (2017) to study height. They are listed in table A.1. Mortality rates are scaled to per 1,000.

Figure A.3: Robustness: How the relationship between birth order and mortality in India differs from the rest of the developing world, restricted sample

(a) Coefficients on *birth order*$_{ims}$ × *India*$_s$ indicators from equation 1, all controls included: NNM, PNM, and IMR are dependent variables

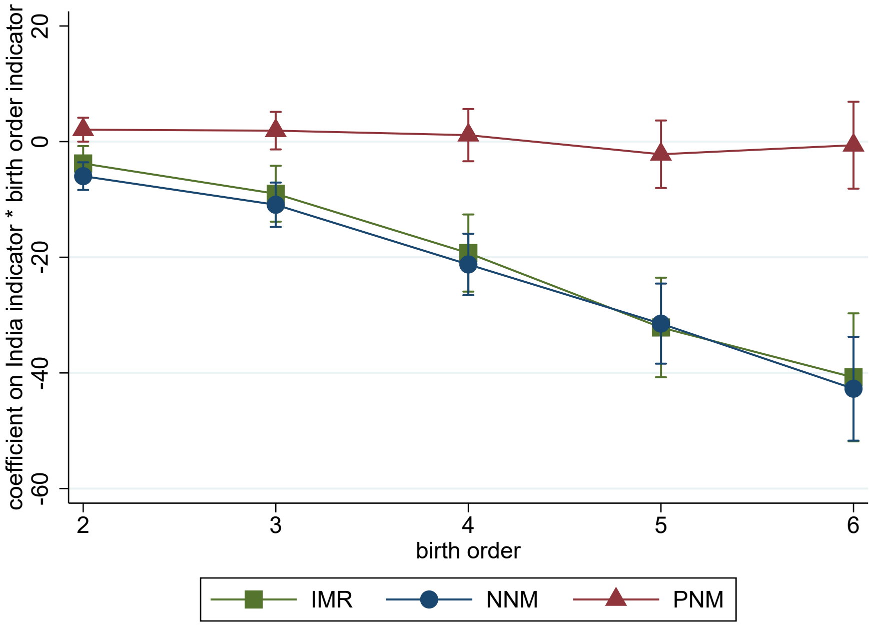

(b) Coefficients on *birth order*$_{ims}$ × *India*$_s$ indicators without sibsize controls, with sibsize controls, and with mother FEs: NNM is the dependent variable

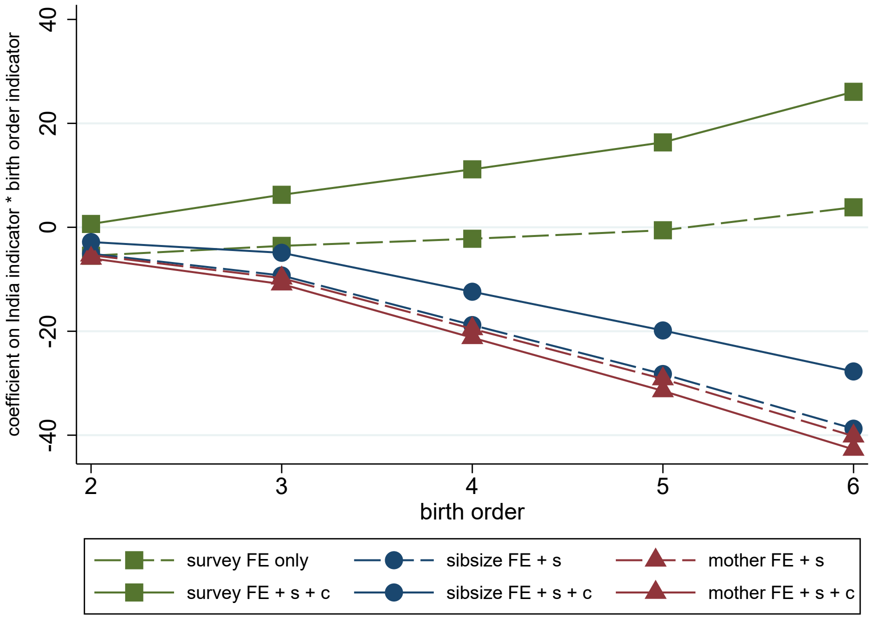

Restricted sample: Starting from the main DHS sample of births described in section 3, this sample excludes all births to mothers who have had a birth in the past five years to avoid confounding by incomplete fertility. Each connected set of estimates is from a separate regression. 95% confidence intervals in panel (a) reflect standard errors clustered by survey PSU. Panel (a) uses the fully controlled specification from equation 1, including mother fixed effects. In panel (b) s = sex and c = birth cohort of mom and child.

Figure A.4: Robustness: How the relationship between birth order and mortality in India differs from an alternative sub-Saharan African comparison sample

(a) Coefficients on *birth order*$_{ims}$ × *India*$_s$ indicators from equation 1, all controls included: NNM, PNM, and IMR are dependent variables

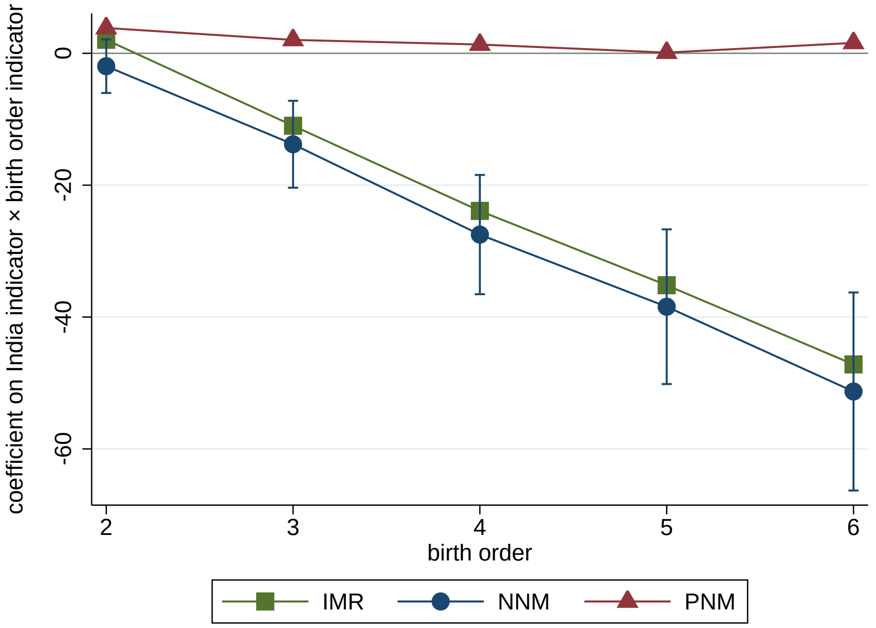

(b) Coefficients on *birth order*$_{ims}$ × *India*$_s$ indicators without sibsize controls, with sibsize controls, and with mother FEs: NNM is the dependent variable

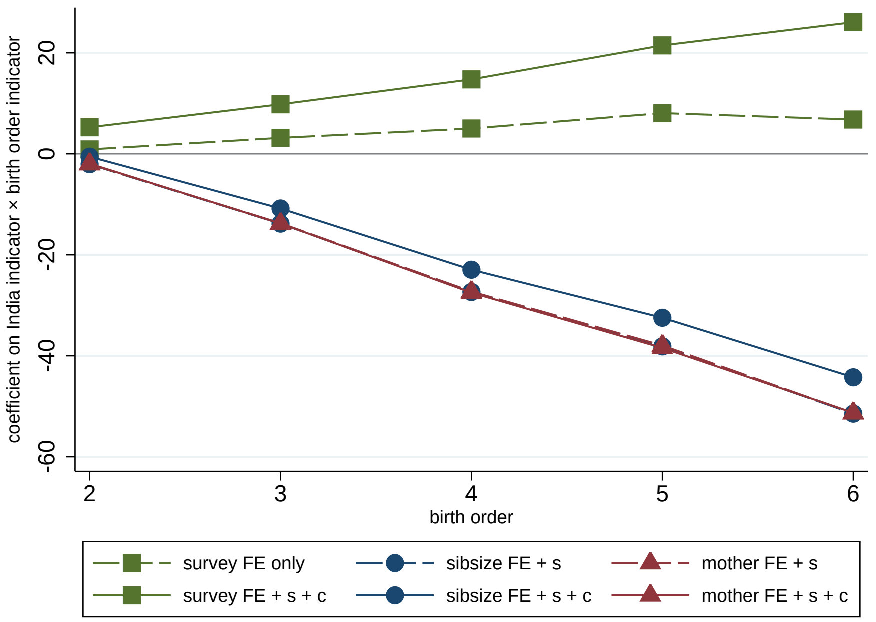

India's 2005-6 DHS is compared with the set of African DHS rounds used by Jayachandran and Pande (2017) to study height and listed in table A.1. Each connected set of estimates is from a separate regression. 95% confidence intervals in panel (a) reflect standard errors clustered by survey PSU. Panel (a) uses the fully controlled specification from equation 1, including mother fixed effects. In panel (b) s = sex and c = birth cohort of mom and child.

Figure A.5: India's birth order pattern of NNM is not reversed by deaths at later ages

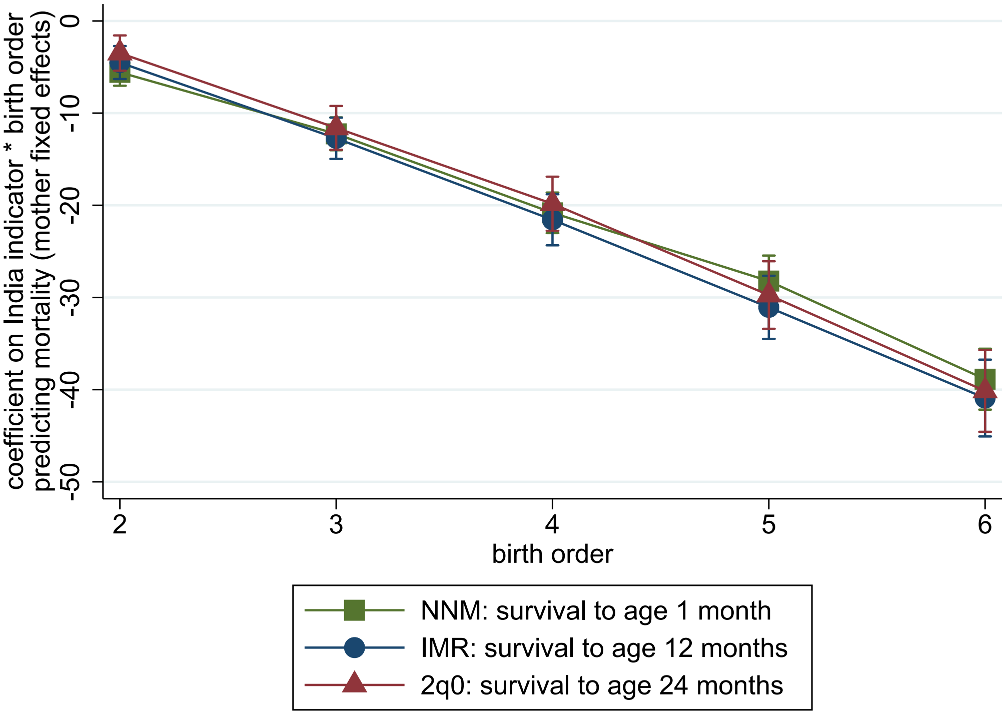

Main DHS sample of births, described in section 3. This figure is a robustness check and extension of panel (a) of figure 2, but with survival to age 2 ($_2q_0$) as a dependent variable. Each mortality rate (NNM, IMR, $_2q_0$) is a dependent variable in a separate regression. The results are slightly quantitatively different from the main result because only children born at least two years before their mother's interview date are included, so that the sample is comparable across the three mortality rates. 95% confidence intervals for the effect on NNM are clustered by survey PSU and overlap with the other coefficients.

Figure A.6: Mothers' BMI in India and in sub-Saharan Africa, by sibsize

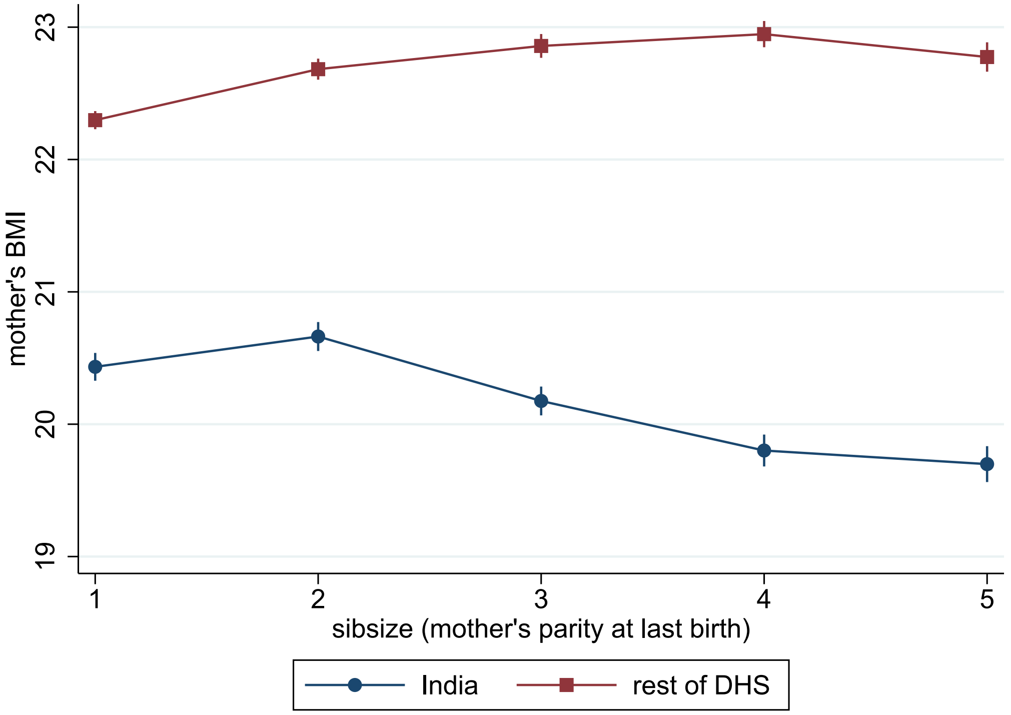

Computations are identical to figure 4 in the main text, but here "rest of DHS" refers to the African comparison sample. We use the same set of DHS surveys used by Jayachandran and Pande (2017) to study height, which is listed in table A.1. Vertical lines are 95% CIs, with standard errors clustered to reflect survey design.

Figure A.7: How age predicts women's BMI, at dichotomised BMI cut-points: India compared with the rest of the DHS

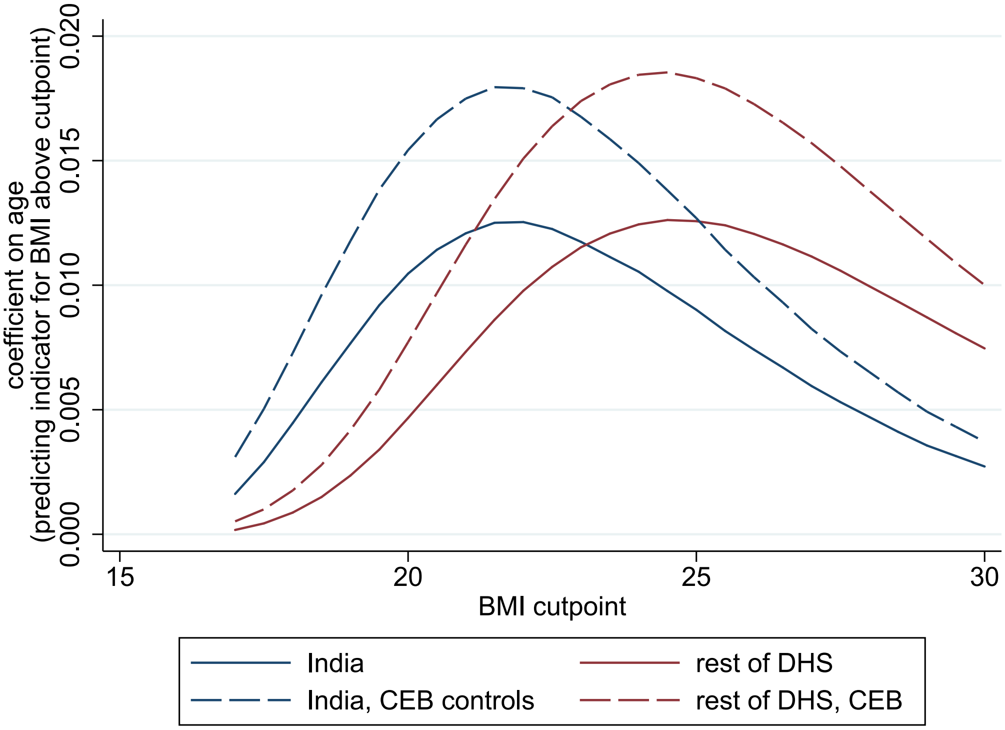

Women's anthropometry sample, described in sections 3 and 6. CEB stands for "children ever born" and indicates that controls for indicators of children ever born at the time of the survey are included. The figure plots and connects coefficients on age estimated from the following linear probability model:
$\mathbf{1}\left[BMI_{ms} > c\right] = \beta_0^s + \beta_1^s \text{age}_{ms} + \theta \text{children ever born}_{ms} + \varepsilon_{ms}$.

Figure A.8: The rate of change in BMI of month-of-birth cohorts of women differs between India and the rest of the DHS

(a) India 1998/9 – 2005/6 is compared to all available DHS

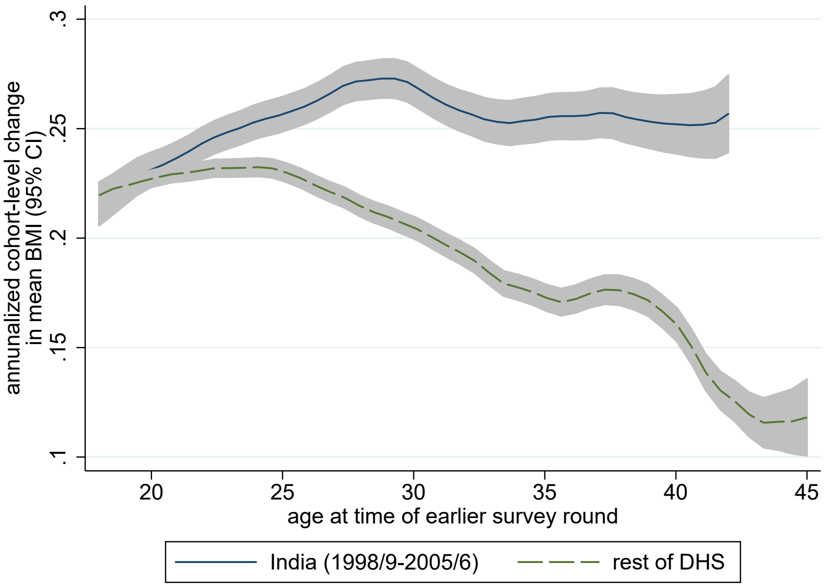

(b) India 1998/9 – 2005/6 is compared to those surveys collected during a similar time period

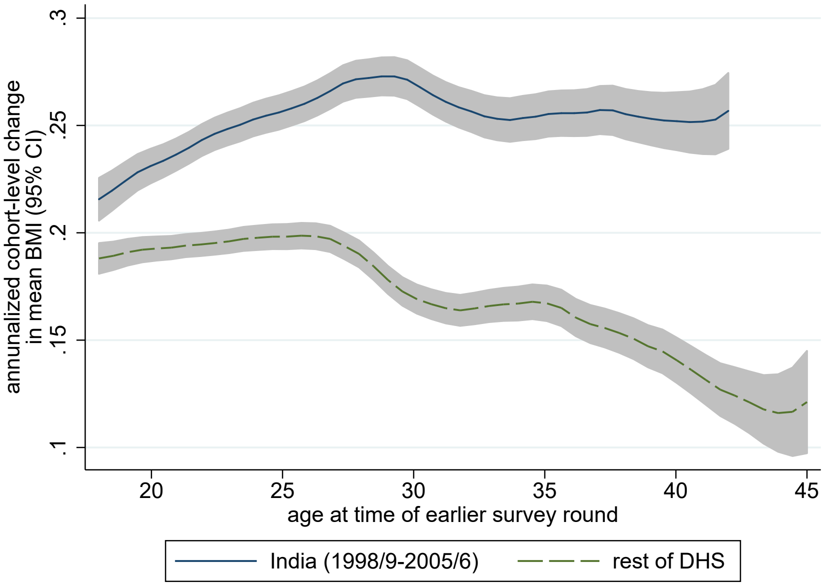

Panel (a) restricts the women's anthropometry sample described in sections 3 and 6 to include those surveys for which there is more than one DHS survey round in the same country. Panel (b) restricts the women's anthropometry sample to those DHS surveys for which the first of two surveys in the same country was within 2.5 years before or after the 1998/9 Indian DHS. In both panels, cohort mean changes are annualised by dividing by the time interval in months between DHS rounds.

Figure A.9: Geographic effect heterogeneity is suggestive of the role of maternal underweight 1: Comparisons within India

Conclusion: The birth order gradient is steeper in north India, where undernutrition is more severe and women's social status is more constrained, than in south India

$$NNM_{im} = \sum_b \beta^b \text{birth order}_{im} + \gamma \text{sex}_{im} + \alpha_m + \varepsilon_{im}.$$

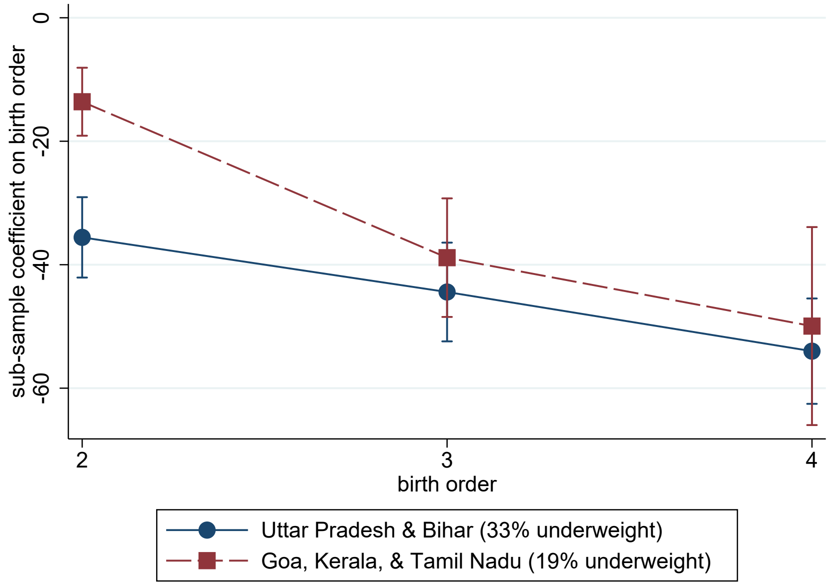

The figure reports two separate mother fixed effects regressions, using data from the listed states from India's 2005-6 DHS.

Figure A.10: Geographic effect heterogeneity is suggestive of the role of maternal underweight 2: Comparisons across full DHS sample

Conclusion: The local fraction of mothers underweight interacts with birth order

$$NNM_{imps} = \sum_b \beta_3^b \textit{birth order}_{imps}^b \times \textit{PSU underweight}_{ps} + \sum_b \beta_2^b \textit{birth order}_{imps}^b + f\left(CMC_{imps}^{child}, \textit{India}_s\right) + \gamma_1 sex_{imps} \times \textit{India}_s + \gamma_2 sex_{imps} + \alpha_{mps} + \varepsilon_{imps}.$$

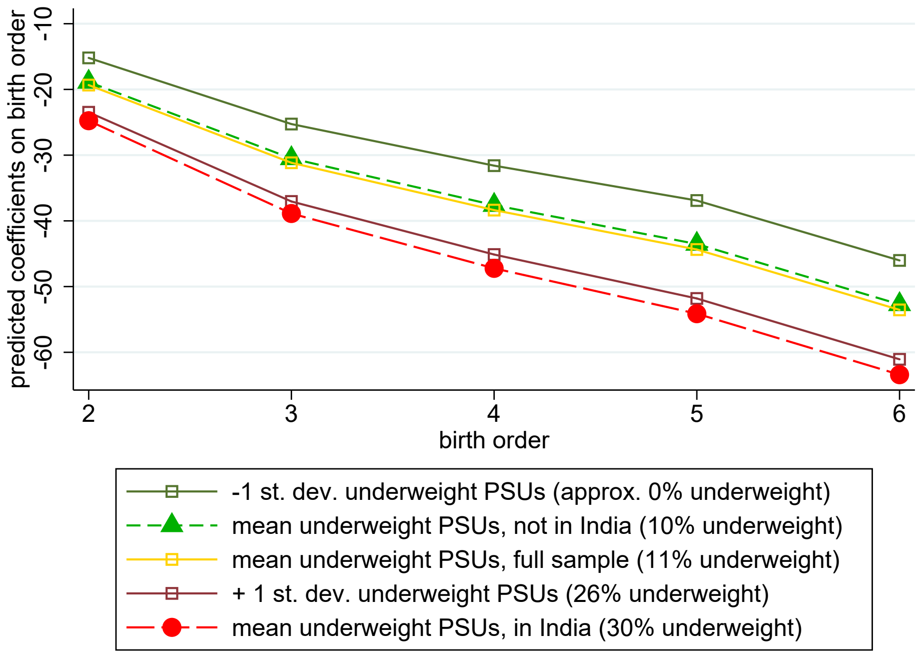

This figure plots predicted effects of being the birth order on the horizontal axis, rather than first-born, at various hypothetical levels of the fraction of women who are underweight in a PSU. This is a way of visualizing the magnitude of the interaction estimated with the equation above. In particular, each dot in the figure is computed as: $\left(\hat{\beta}_3^b \times \textit{PSU underweight} + \hat{\beta}_2^b\right)$ at each birth order b, for a specified example levels of *PSU underweight*.

The data with which the regression equation is computed for the main DHS sample. Note that birth order is not interacted with India in the regression equation. Instead, the figure includes among the example hypothetical levels of *PSU underweight* the mean within India and the mean for our data outside of India; comparing these two lines would offer a linear post-diction of the interaction between India and birth order. PSU underweight is the fraction, in a local area, of all women of childbearing age measured by the DHS who have a BMI below 18.5; women are included whether or not they have given birth, and each woman is equally weighted in the mean, regardless of how many times she has given birth. PSU = primary sampling unit.

Figure A.11: Across DHS survey rounds, a larger negative effect of birth order on NNM is associated with a steeper negative gradient between age and underweight among adult women (plots of t-statistics and F-statistics)

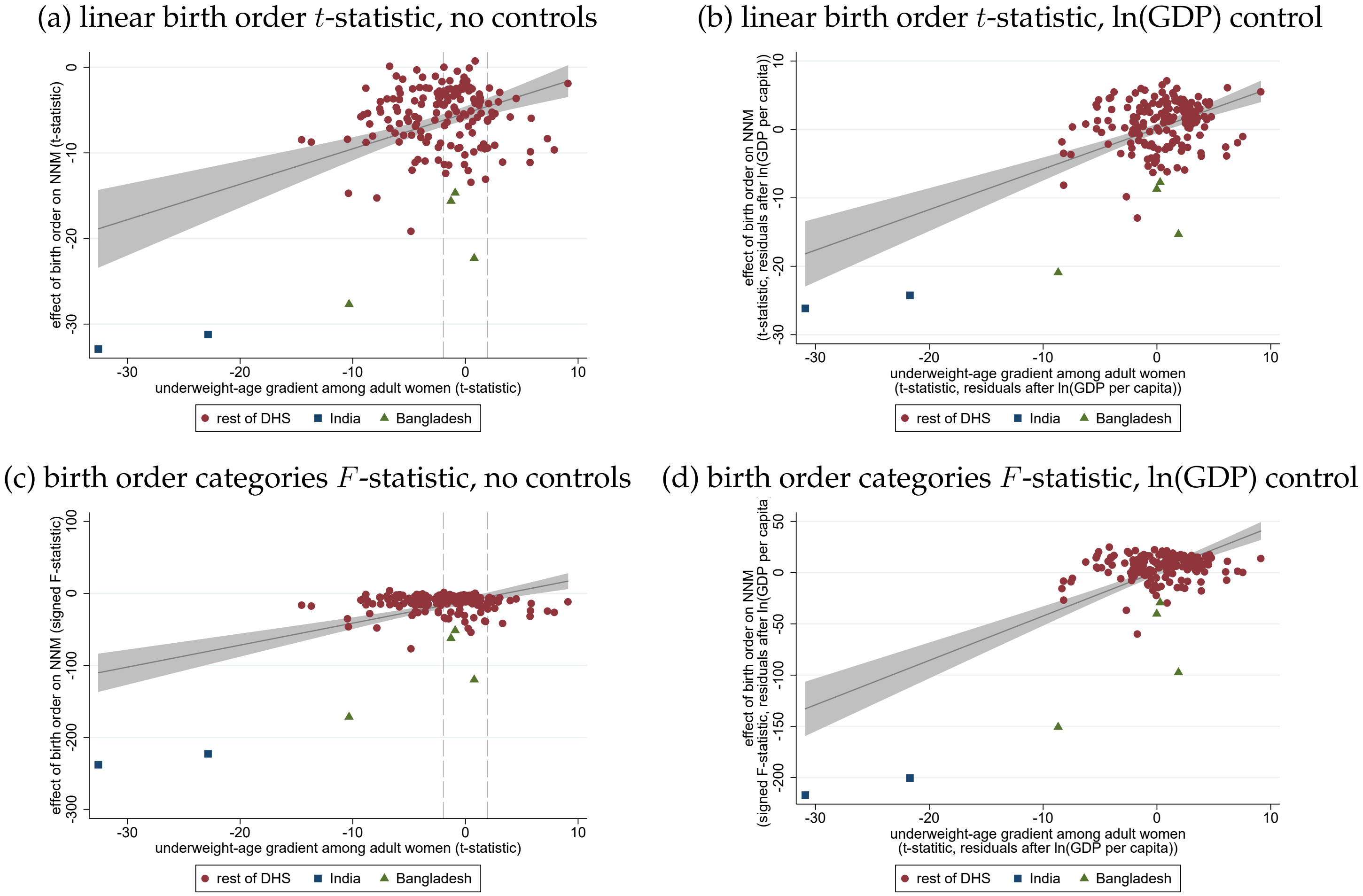

The sample is DHS survey rounds used to construct the main DHS sample of births discussed in section 3. In all panels, each point plots regression results from two separate regressions, estimated for one DHS survey round at a time. The horizontal axis in all panels plots the t-statistics from the same coefficients in the regressions of underweight on the age of adult women in figure 7. In panels (a) and (b), the vertical axis plots the t-statistics on the linear birth order coefficient from the regressions in figure 7 of NNM on birth order, entered linearly, with mother fixed effects. In panels (c) and (d), the vertical axis plots F-statistics (multiplied by the sign of the linear birth order coefficient) from a joint test of all birth order indicators in a regression of NNM on birth order indicators (instead of birth order as a linear independent variable) in regressions with mother fixed effects. The results in panels (b) and (d) additionally control (by residualizing the variables in the horizontal and vertical axes in two separate regressions) for a DHS-survey-round-level mean of GDP per capita; for more detail see section 6.5.

Table A.1: Demographic and Health Surveys in each sample

country	v000	year	main	SSA	longitudinal	long.-restricted
Albania	AL5	2008-09	✓			
Armenia	AM4	2000, 2005	✓		✓	✓
Armenia	AM7	2015-2016	✓		✓	✓
Azerbaijan	AZ5	2006	✓			
Bangladesh	BD3	1996-97, 1999-2000	✓		✓	✓
Bangladesh	BD4	2004	✓		✓	✓
Bangladesh	BD5	2007	✓		✓	✓
Bangladesh	BD6	2011, 2014	✓		✓	✓
Benin	BJ3	1996	✓		✓	✓
Benin	BJ4	2001	✓		✓	✓
Benin	BJ5	2006	✓		✓	✓
Benin	BJ6	2011-12	✓		✓	✓
Bolivia	BO3	1993-94, 1998	✓		✓	✓
Bolivia	BO4	2003-04	✓		✓	✓
Bolivia	BO5	2008	✓		✓	✓
Brazil	BR3	1996	✓			
Burkina Faso	BF2	1992-93	✓		✓	
Burkina Faso	BF3	1998-99	✓		✓	✓
Burkina Faso	BF4	2003	✓		✓	✓
Burkina Faso	BF6	2010	✓		✓	✓
Burundi	BU6	2010	✓			
Cambodia	KH4	2000	✓		✓	✓
Cambodia	KH5	2005-06, 2010-11	✓		✓	✓
Cambodia	KH6	2014	✓		✓	✓
Cameroon	CM3	1998	✓		✓	✓
Cameroon	CM4	2004	✓	✓	✓	✓
Cameroon	CM6	2011	✓		✓	✓
Central African Republic	CF3	1994-95	✓			
Chad	TD3	1996-97	✓		✓	✓
Chad	TD4	2004	✓	✓	✓	✓
Chad	TD6	2014-2015	✓		✓	✓
Colombia	CO3	1995	✓		✓	
Colombia	CO4	2000, 2004-05	✓		✓	✓
Colombia	CO5	2009-10	✓		✓	✓
Comoros	KM3	1996	✓		✓	
Comoros	KM6	2012	✓		✓	
Congo, Democratic Republic	CD5	2007	✓	✓	✓	
Congo, Democratic Republic	CD6	2013-14	✓		✓	
Cote d'Ivoire	CI3	1994, 1998-99	✓		✓	✓
Cote d'Ivoire	CI6	2011-12	✓		✓	✓
Dominican Republic	DR2	1991	✓		✓	
Dominican Republic	DR3	1996	✓		✓	✓
Dominican Republic	DR6	2013	✓		✓	✓
Egypt	EG2	1992-93	✓		✓	
Egypt	EG3	1995-96	✓		✓	
Egypt	EG4	2000, 2003, 2005	✓		✓	✓
Egypt	EG5	2008	✓		✓	✓
Egypt	EG6	2014	✓		✓	✓

Table A.1: Demographic and Health Surveys in each sample

country	v000	year	main	SSA	longitudinal	long.-restricted
Ethiopia	ET4	2000, 2005	✓	✓	✓	✓
Ethiopia	ET6	2011	✓		✓	✓
Ethiopia	ET7	2016	✓		✓	✓
Gabon	GA3	2000	✓		✓	✓
Gabon	GA6	2012	✓		✓	✓
Gambia	GM6	2013	✓			
Ghana	GH2	1993-94	✓		✓	
Ghana	GH3	1998-99	✓		✓	✓
Ghana	GH4	2003	✓		✓	✓
Ghana	GH5	2008	✓	✓	✓	✓
Ghana	GH6	2014	✓		✓	✓
Guatemala	GU3	1995, 1998-99	✓		✓	✓
Guatemala	GU6	2014-15	✓		✓	✓
Guinea	GN3	1999	✓		✓	✓
Guinea	GN4	2005	✓	✓	✓	✓
Guinea	GN6	2012	✓		✓	✓
Guyana	GY5	2009	✓			
Haiti	HT3	1994-95	✓		✓	
Haiti	HT4	2000	✓		✓	✓
Haiti	HT5	2005-06	✓		✓	✓
Haiti	HT6	2012	✓		✓	✓
Honduras	HN5	2005-06	✓		✓	
Honduras	HN6	2011-12	✓		✓	
India	IA2	1992-93	✓			
India	IA3	1998-99	✓		✓	✓
India	IA5	2005-06	✓	✓	✓	✓
Jordan	JO3	1997	✓		✓	✓
Jordan	JO4	2002	✓		✓	✓
Jordan	JO5	2007, 2009	✓		✓	
Jordan	JO6	2012	✓		✓	
Kazakhstan	KK3	1995, 1999	✓			
Kenya	KE2	1993	✓		✓	
Kenya	KE3	1998	✓		✓	✓
Kenya	KE4	2003	✓		✓	✓
Kenya	KE5	2008-09	✓	✓	✓	
Kenya	KE6	2014	✓		✓	
Kyrgyz Republic	KY3	1997	✓		✓	✓
Kyrgyz Republic	KY6	2012	✓		✓	✓
Lesotho	LS4	2004	✓	✓	✓	
Lesotho	LS5	2009	✓	✓	✓	
Lesotho	LS6	2014	✓		✓	
Liberia	LB5	2007	✓	✓	✓	
Liberia	LB6	2013	✓		✓	
Madagascar	MD3	1997	✓		✓	✓
Madagascar	MD4	2003-04	✓	✓	✓	✓
Madagascar	MD5	2008-09	✓		✓	✓
Malawi	MW2	1992	✓		✓	
Malawi	MW4	2000, 2004-05	✓	✓	✓	✓

Table A.1: Demographic and Health Surveys in each sample

country	v000	year	main	SSA	longitudinal	long.-restricted
Malawi	MW5	2010	✓		✓	✓
Malawi	MW7	2015-16	✓		✓	✓
Maldives	MV5	2009	✓			
Mali	ML3	1995-96	✓		✓	
Mali	ML4	2001	✓		✓	✓
Mali	ML5	2006	✓	✓	✓	✓
Mali	ML6	2012-13	✓		✓	✓
Moldova	MB4	2005	✓			
Morocco	MA2	1992	✓		✓	
Morocco	MA4	2003-04	✓		✓	
Mozambique	MZ3	1997	✓		✓	✓
Mozambique	MZ4	2003-04	✓		✓	✓
Mozambique	MZ6	2011	✓		✓	✓
Namibia	NM2	1992	✓		✓	
Namibia	NM5	2006-07	✓	✓	✓	
Namibia	NM6	2013	✓		✓	
Nepal	NP3	1996	✓		✓	
Nepal	NP4	2001	✓		✓	
Nepal	NP5	2006	✓		✓	
Nepal	NP6	2011	✓		✓	
Nicaragua	NC3	1998	✓		✓	✓
Nicaragua	NC4	2001	✓		✓	✓
Niger	NI2	1992	✓		✓	
Niger	NI3	1998	✓		✓	✓
Niger	NI5	2006-07	✓	✓	✓	✓
Niger	NI6	2012	✓		✓	✓
Nigeria	NG4	2003	✓		✓	
Nigeria	NG5	2008	✓	✓	✓	
Nigeria	NG6	2013	✓		✓	
Pakistan	PK6	2012-13	✓			
Peru	PE2	1991-92	✓		✓	
Peru	PE3	1996	✓		✓	✓
Peru	PE4	2000	✓		✓	✓
Peru	PE5	2004-08	✓		✓	✓
Peru	PE6	2009, 2010, 2011	✓		✓	✓
Republic of Congo	CG5	2005	✓	✓	✓	
Republic of Congo	CG6	2011-2012	✓		✓	
Rwanda	RW4	2000, 2005	✓	✓	✓	✓
Rwanda	RW6	2010-11, 2014-15	✓		✓	✓
Sao Tome and Principe	ST5	2008-09	✓	✓		
Senegal	SN2	1992-93	✓		✓	
Senegal	SN4	2005	✓	✓	✓	
Senegal	SN6	2010-11	✓		✓	
Sierra Leone	SL5	2008	✓	✓	✓	
Sierra Leone	SL6	2013	✓		✓	
Swaziland	SZ5	2006-07	✓	✓		
Tajikistan	TJ6	2012	✓			
Tanzania	TZ2	1991-92	✓		✓	

Table A.1: Demographic and Health Surveys in each sample

country	v000	year	main	SSA	longitudinal	long.-restricted
Tanzania	TZ3	1996	✓		✓	✓
Tanzania	TZ4	2004-05	✓	✓	✓	✓
Tanzania	TZ5	2009-10	✓	✓	✓	✓
Tanzania	TZ7	2015-16	✓		✓	✓
Timor-Leste	TL5	2009-10	✓			
Togo	TG3	1998	✓		✓	✓
Togo	TG6	2013-14	✓		✓	✓
Turkey	TR2	1993	✓		✓	
Turkey	TR3	1998	✓		✓	✓
Turkey	TR4	2003-04	✓		✓	✓
Uganda	UG3	1995	✓		✓	
Uganda	UG4	2000-01	✓		✓	✓
Uganda	UG5	2006	✓	✓	✓	✓
Uganda	UG6	2011	✓		✓	✓
Uzbekistan	UZ3	1996	✓			
Yemen	YE6	2013	✓			
Zambia	ZM2	1992	✓		✓	
Zambia	ZM3	1996-97	✓		✓	✓
Zambia	ZM4	2001-02	✓		✓	✓
Zambia	ZM5	2007	✓	✓	✓	✓
Zambia	ZM6	2013-14	✓		✓	✓
Zimbabwe	ZW3	1994	✓		✓	
Zimbabwe	ZW4	1999	✓		✓	✓
Zimbabwe	ZW5	2005-06	✓	✓	✓	✓
Zimbabwe	ZW6	2010-11	✓		✓	✓

Each row is one of 169 survey rounds of the Demographic and Health Surveys. We include in our main DHS sample of births (marked "main") all DHS rounds that measured maternal anthropometry plus the three Indian DHS. "SSA" indicates the replication sample that compares India with sub-Saharan Africa (such as in figure A.2; this set of DHS rounds matches that used to study height by Jayachandran and Pande, 2017 and Spears, 2017). The longitudinal and longitudinal-restricted samples are used in panels (a) and (b), respectively, of figures 6 and A.8; surveys are excluded if there is only one round per country with adult women's anthropometry. For the reader's convenience, we include v000, which is the code for a DHS survey round provided with the data. All data are publicly available free of charge at measuredhs.com.

Table A.2: Effects of birth order on NNM, India vs. rest of DHS

dependent variable:	(1) NNM	(2) NNM	(3) NNM	(4) NNM	(5) NNM	(6) NNM	(7) NNM	(8) NNM	(9) NNM
birth order 2 × India	-4.363***	-3.787***	0.364	-5.591***	-3.477***	-6.528***	-3.478***	-5.570***	-6.065***
	(0.654)	(0.654)	(0.678)	(0.706)	(0.749)	(0.725)	(0.749)	(0.707)	(0.937)
birth order 3 × India	-4.075***	-3.533***	3.952***	-13.07***	-9.138***	-14.88***	-9.118***	-13.03***	-14.05***
	(0.750)	(0.754)	(0.834)	(0.865)	(1.006)	(0.925)	(1.006)	(0.866)	(1.488)
birth order 4 × India	-1.907*	-1.271	9.805***	-21.48***	-15.86***	-24.12***	-15.82***	-21.39***	-23.03***
	(0.898)	(0.904)	(1.047)	(1.068)	(1.298)	(1.166)	(1.300)	(1.068)	(2.055)
birth order 5 × India	0.385	1.269	15.98***	-29.57***	-22.33***	-33.03***	-22.28***	-29.38***	-31.69***
	(1.126)	(1.131)	(1.332)	(1.349)	(1.645)	(1.474)	(1.646)	(1.349)	(2.662)
birth order 6+ × India	2.886*	4.128**	24.73***	-39.31***	-29.73***	-43.97***	-29.70***	-39.17***	-41.77***
	(1.281)	(1.301)	(1.627)	(1.599)	(2.000)	(1.784)	(2.000)	(1.600)	(3.450)
birth order 2	-10.02***	-9.167***	-5.891***	-14.68***	-13.04***	-15.87***	-13.03***	-14.67***	-18.80***
	(0.211)	(0.211)	(0.221)	(0.230)	(0.246)	(0.238)	(0.246)	(0.230)	(0.278)
birth order 3	-11.91***	-10.39***	-4.129***	-21.92***	-18.68***	-24.26***	-18.67***	-21.91***	-30.03***
	(0.239)	(0.239)	(0.268)	(0.272)	(0.320)	(0.297)	(0.320)	(0.272)	(0.411)
birth order 4	-10.47***	-8.316***	0.629+	-24.96***	-20.22***	-28.35***	-20.21***	-24.94***	-36.75***
	(0.271)	(0.273)	(0.324)	(0.315)	(0.399)	(0.359)	(0.399)	(0.315)	(0.547)
birth order 5	-8.711***	-5.867***	5.635***	-27.08***	-20.91***	-31.46***	-20.89***	-27.07***	-42.38***
	(0.315)	(0.318)	(0.390)	(0.366)	(0.484)	(0.427)	(0.484)	(0.366)	(0.688)
birth order 6+	-0.636*	3.766***	20.42***	-29.15***	-20.17***	-35.46***	-20.14***	-29.13***	-51.18***
	(0.316)	(0.324)	(0.452)	(0.388)	(0.574)	(0.490)	(0.574)	(0.388)	(0.881)
survey round fixed effects	✓	✓	✓	✓	✓	✓	✓	✓	✓
child sex and birth cohort		✓	✓	✓	✓	✓	✓	✓	✓
mother birth cohort			✓		✓		✓		✓
sibsize				✓	✓				
sibling sex combinations						✓	✓		
mother fixed effects								✓	✓
n (live births)	6,695,004	6,695,004	6,695,004	6,695,004	6,695,004	6,695,004	6,695,004	6,339,396	6,339,396

Main DHS sample of births, described in section 3. The results in column 9 of this table are plotted in panel (b) of figure 2. NNM = neonatal mortality. Observations are live births that occurred at least 1 month before the interview date. Mother and child cohorts are cubic polynomials of CMC code of month of birth. All controls are fully interacted with an India indicator. Standard errors clustered by DHS PSU.

Table A.3: Effects of birth order on IMR, India vs. rest of DHS

	(1)	(2)	(3)	(4)	(5)	(6)	(7)	(8)	(9)
dependent variable:	IMR	IMR	IMR	IMR	IMR	IMR	IMR	IMR	IMR
birth order 2 × India	-4.402***	-3.659***	0.427	-4.996***	-2.947**	-5.385***	-2.939**	-4.378***	-2.661*
	(0.825)	(0.828)	(0.864)	(0.877)	(0.936)	(0.898)	(0.936)	(0.887)	(1.184)
birth order 3 × India	-3.768***	-3.260***	3.871***	-13.93***	-10.24***	-14.72***	-10.22***	-12.85***	-9.436***
	(0.975)	(0.980)	(1.082)	(1.100)	(1.273)	(1.165)	(1.275)	(1.110)	(1.909)
birth order 4 × India	1.575	1.979+	12.75***	-23.07***	-17.80***	-24.31***	-17.82***	-21.71***	-16.74***
	(1.184)	(1.194)	(1.372)	(1.370)	(1.658)	(1.481)	(1.660)	(1.379)	(2.632)
birth order 5 × India	5.153***	5.676***	20.24***	-33.04***	-26.20***	-34.71***	-26.25***	-31.47***	-25.02***
	(1.462)	(1.474)	(1.727)	(1.699)	(2.070)	(1.836)	(2.072)	(1.707)	(3.357)
birth order 6+ × India	12.06***	12.36***	32.32***	-41.79***	-33.53***	-44.00***	-33.65***	-40.68***	-31.16***
	(1.717)	(1.745)	(2.153)	(2.061)	(2.572)	(2.258)	(2.573)	(2.070)	(4.372)
birth order 2	-10.82***	-8.870***	-0.949**	-20.04***	-14.22***	-21.48***	-14.21***	-19.38***	-22.39***
	(0.299)	(0.300)	(0.315)	(0.320)	(0.341)	(0.329)	(0.341)	(0.324)	(0.392)
birth order 3	-12.47***	-8.948***	6.216***	-32.48***	-21.26***	-35.38***	-21.24***	-31.68***	-37.68***
	(0.341)	(0.343)	(0.385)	(0.380)	(0.446)	(0.410)	(0.446)	(0.384)	(0.579)
birth order 4	-10.10***	-5.088***	16.63***	-40.23***	-24.06***	-44.49***	-24.04***	-39.43***	-48.20***
	(0.392)	(0.396)	(0.468)	(0.445)	(0.560)	(0.499)	(0.560)	(0.449)	(0.774)
birth order 5	-7.150***	-0.539	27.42***	-46.49***	-25.64***	-52.05***	-25.61***	-45.80***	-57.23***
	(0.456)	(0.460)	(0.561)	(0.520)	(0.680)	(0.596)	(0.680)	(0.524)	(0.973)
birth order 6+	4.736***	14.93***	55.37***	-56.55***	-26.65***	-64.61***	-26.61***	-55.93***	-72.43***
	(0.452)	(0.461)	(0.641)	(0.558)	(0.806)	(0.681)	(0.806)	(0.562)	(1.239)
survey round fixed effects	✓	✓	✓	✓	✓	✓	✓	✓	✓
child sex and birth cohort		✓	✓	✓	✓	✓	✓	✓	✓
mother birth cohort			✓		✓		✓		✓
sibsize indicators				✓	✓				
sibling sex combinations						✓	✓		
mother fixed effects								✓	✓
n (live births)	6,373,337	6,373,337	6,373,337	6,373,337	6,373,337	6,373,337	6,373,337	6,030,617	6,030,617

Main DHS sample of births, described in section 3. Regressions and sample correspond with figure 2, where panel (a) plots column 9. IMR = infant mortality. Observations are live births in the DHS birth history at least 1 year before the interview date. Mother and child cohorts are cubic polynomials of CMC code of month of birth. All controls are fully interacted with an India indicator. Standard errors clustered by DHS PSU.

Table A.4: The effect of birth order on NNM is robust to controlling for sibsize × India × own sex indicators

	(1)	(2)
dependent variable:	NNM	NNM
birth order 2 × India	-6.07***	-6.05***
	(0.94)	(0.94)
birth order 3 × India	-14.05***	-13.90***
	(1.49)	(1.49)
birth order 4 × India	-23.03***	-22.94***
	(2.06)	(2.05)
birth order 5 × India	-31.69***	-31.76***
	(2.66)	(2.66)
birth order 6+ × India	-41.77***	-42.12***
	(3.45)	(3.45)
non-interacted birth order indicators	✓	✓
survey round fixed effects	✓	✓
child sex and birth cohort (cubic) × India	✓	✓
mother birth cohort (cubic) × India	✓	✓
mother fixed effects	✓	✓
sibsize × India × child own sex		✓
n (live births)	6,339,396	6,339,396

Main DHS sample of births, described in section 3. NNM = neonatal mortality. Observations are live births that occurred at least 1 month before the interview date. Mother and child cohorts are cubic polynomials of CMC code of month of birth. All controls are fully interacted with an India indicator. Standard errors clustered by DHS PSU. The sample in this table is smaller than the sample in table A.2, which presents similar results, because the fixed effects are finer and observations are omitted in fixed effect categories with no within variation.

Table A.5: Institutional delivery: Regression results for children under 5

	(1)	(2)	(3)	(4)	(5)	(6)	(7)
sample:	full	columns 2-7 include only children under 5 with recorded delivery place					
dependent variable:	NNM	NNM	NNM	NNM	NNM	institutional	institutional
2nd born	-18.87***	-42.60***	-42.62***	-47.17***	-46.77***	-0.0130*	-0.0970***
	(1.105)	(3.368)	(3.368)	(3.571)	(3.566)	(0.00611)	(0.00672)
3rd born	-35.92***	-87.99***	-87.97***	-97.73***	-97.15***	0.0134	-0.143***
	(1.437)	(5.524)	(5.526)	(6.053)	(6.033)	(0.00981)	(0.0115)
4th born	-48.86***	-125.0***	-124.9***	-140.3***	-139.7***	0.0561***	-0.159***
	(1.856)	(8.537)	(8.552)	(9.355)	(9.311)	(0.0135)	(0.0164)
5th born	-63.26***	-168.2***	-168.1***	-190.5***	-189.8***	0.0938***	-0.175***
	(2.625)	(11.72)	(11.75)	(12.80)	(12.72)	(0.0174)	(0.0214)
6th born	-73.91***	-186.3***	-186.2***	-217.2***	-216.4***	0.134***	-0.185***
	(3.828)	(14.56)	(14.59)	(15.80)	(15.74)	(0.0220)	(0.0270)
institutional			-1.158		4.064†		
			(1.887)		(2.081)		
sibsize indicators	✓	✓	✓	✓	✓	✓	✓
further controls				✓	✓		✓
n	221,743	48,156	48,156	48,156	48,156	48,156	48,156

Data are India's 2005-6 DHS, corresponding with figure 3. Standard errors clustered by survey PSU. "Institutional" is an indicator for institutional delivery, rather than delivery at a home. "Further controls" are the century-month code of the birth cohort of the child and the mother (both entered as quadratic polynomials), the sex of the child, and whether the child lives in an urban or rural place.

Note on sample restriction: The results in column 1 use a sample that includes all available births from the 2005-6 Indian DHS. These results are included for comparison because the results in columns 2-7 necessarily use a restricted sample of children under 5 years old because these are the children for whom place of birth was recorded. This sample restriction complicates the identification of birth order effects, even if sibsize is controlled for. This is because birth order, sibsize, and birth spacing jointly predict selection into the sample (Spears et al., 2019). Consider, for example, a child of birth order 1 in a sibsize of 3: such a child will only be under 5 at the time of the survey if his or her mother has had three children in less than five years, and therefore if he or she comes from a household with low birth spacing. In contrast, a child of birth order 2 in a sibsize of 3 would be expected to have longer birth spacing than the first child, and a child of birth order 3 could have been born at any point in the five year period with any birth spacing. So, part of what appears to be a birth order effect in this sample is, in fact, a household composition effect of selection into the sample. This is why the birth order gradient is steeper here (and in columns 2-5) than in the main result (or, comparably, in column 1). Our point in including this analysis is merely to verify that *institutional delivery* is not an omitted variable in our results: it is not predicted by birth order, and controlling for it does not change the coefficient on birth order predicting NNM.

Table A.6: Following Lundberg and Svaleryd (2017), we find that excluding possible-replacement last births (after prior sibling NNM) preserves the pattern

	(1)	(2)
dependent variable:	NNM	NNM
excluded:	last-borns w/prior sibling death	last-borns w/prior sibling NNM
birth order 2 × India	-4.028***	-4.078***
	(0.679)	(0.676)
birth order 3 × India	-6.860***	-6.841***
	(0.834)	(0.821)
birth order 4 × India	-10.08***	-9.460***
	(1.067)	(1.026)
birth order 5 × India	-12.66***	-10.92***
	(1.400)	(1.311)
birth order 6 × India	-26.19***	-23.22***
	(1.711)	(1.606)
mother FEs & controls	✓	✓
n	6,066,288	6,227,729
NNM among included	38.54	38.07
NNM among excluded	69.52	140.7

The sample starts from the main "India vs. rest of DHS" sample in Table A.2, but excludes last-born children (where "last-born" is at the time of the survey, within a sibship) whose prior sibling has died (either at a neonatal age or at any age, according to the column header). This robustness check is intended to rule out the biasing threat of endogenous fertility, where mothers would be more likely to have a "replacement" birth after the death of a prior child. Note that because this analysis uses mother fixed effects, we do not use an explicit sibsize variable, so this last-born exclusion does not require a counterfactual sibsize (recall also that controlling for sibship size and sex structure rather than mother fixed effects did not change our main result). This analysis follows that of Lundberg and Svaleryd (2017), who use it to investigate the possible threat of endogenous fertility in a study of birth order in Swedish data.

Note that, although our effect remains visible with these births (and deaths) excluded from the sample — suggesting that endogenous fertility does not drive our result in this way — this is not the type of robustness check where we would expect the coefficient estimates to be quantitatively unchanged. That is because there is unobserved heterogeneity in the "frailty" of children, for reasons that would be correlated within sibships but orthogonal to birth order within a sibship (such as the sanitation and disease environment of a village). By excluding children whose sibling has died, we are reducing the average frailty of our sample. Thus, in the last row of the table, NNM is higher among excluded births than among included births.

Lundberg and Svaleryd report results excluding *all* children who are last-born to their mothers. Although not reported in this table (but available in the replication files), our results are robust to using this sample (with about 4.7 million observations): the coefficients on birth order × India are numerically similar to our main results: -8 for second-born, -15 for third-born, -22 for fouth-born, etc. Such a robustness check, unlike those reported in this table, does not selectively exclude children of high-frailty sibships.

Table A.7: India's later-born NNM advantage is seen for sibships with first-born boys and first-born girls, but is stronger in sibships of first-born boys

	(1)	(2)	(3)	(4)	(5)	(6)
sex of first-born to mother:	boy	girl	boy	girl	boy	girl
birth order 2 × India	-7.80***	-1.19	-7.17***	-0.72	-7.81***	-1.14
	(1.15)	(1.07)	(1.15)	(1.07)	(1.15)	(1.07)
birth order 3 × India	-18.31***	-5.65***	-16.96***	-4.71***	-18.28***	-5.61***
	(1.39)	(1.23)	(1.40)	(1.24)	(1.39)	(1.23)
birth order 4 × India	-31.67***	-9.51***	-29.74***	-8.16***	-31.69***	-9.45***
	(1.64)	(1.49)	(1.66)	(1.50)	(1.64)	(1.49)
birth order 5 × India	-39.55***	-17.64***	-37.13***	-15.93***	-39.68***	-17.62***
	(2.05)	(1.83)	(2.07)	(1.86)	(2.05)	(1.83)
birth order 2 × India	-49.60***	-26.96***	-45.30***	-23.97***	-49.71***	-26.85***
	(2.43)	(2.10)	(2.46)	(2.14)	(2.43)	(2.10)
birth order 2	-16.76***	-11.72***	-21.92***	-15.00***	-16.72***	-11.78***
	(0.36)	(0.34)	(0.42)	(0.40)	(0.36)	(0.34)
birth order 3	-25.23***	-17.67***	-35.38***	-24.13***	-25.19***	-17.73***
	(0.42)	(0.39)	(0.60)	(0.57)	(0.42)	(0.39)
birth order 4	-28.94***	-20.00***	-43.68***	-29.39***	-28.90***	-20.08***
	(0.48)	(0.45)	(0.79)	(0.75)	(0.48)	(0.45)
birth order 5	-31.85***	-21.30***	-50.97***	-33.49***	-31.82***	-21.35***
	(0.54)	(0.52)	(0.98)	(0.94)	(0.54)	(0.52)
birth order 6	-33.87***	-23.40***	-61.41***	-40.97***	-33.84***	-23.46***
	(0.58)	(0.54)	(1.24)	(1.19)	(0.58)	(0.54)
n (live births)	3,238,725	3,100,671	3,238,725	3,100,671	3,422,689	3,272,315
child sex	✓	✓	✓	✓	✓	✓
mother fixed effects	✓	✓	✓	✓		
child birth cohort			✓	✓		
sibling sex combinations					✓	✓

The data are the main "India vs. rest of DHS" sample in Table A.2. Note that the sum of the sample sizes in columns 1 and 2 or 3 and 4 of this table match the sample size in columns 8 and 9 (which have mother fixed effects) of Table A.2 ($3,238,725 + 3,100,671 = 6,339,396$). Child cohort is a cubic polynomial of CMC code of month of birth. All controls are fully interacted with an India indicator. Standard errors clustered by DHS PSU.

Note that sex-selective abortion is uncommon for first-born children: even in India, where son preference shapes fertility stopping behavior, the sex of the first born is generally taken to be random (Clark, 2000). So, the sex of the first-born child is not a choice variable. However, subsequent choices, such as the decision to have an additional child, may be correlated with the sex of the first child, so that, for example, Indian mothers who have 3 children rather than 2 are poorer, on average, if they had a first boy than if they had a first girl; this example of heterogeneity, however, would be absorbed by mother fixed effects.
